# Root exudates protect rhizosphere *Pseudomonas* from water stress

**DOI:** 10.1128/aem.00768-25

**Published:** 2025-08-05

**Authors:** Ankita Bhattacharyya, Clint D. Pablo, Olga V. Mavrodi, Alex S. Flynt, David M. Weller, Linda S. Thomashow, Dmitri V. Mavrodi

**Affiliations:** 1 School of Biological, Environmental, and Earth Sciences, The University of Southern Mississippi, Hattiesburg, Mississippi, USA; 2 Department of Plant Pathology, Washington State University 744660, Pullman, Washington, USA; 3 Department of Biomedical Engineering, University of Mississippi 8083, University, Mississippi, USA; 4 USDA Agricultural Research Service, Wheat Health, Genetics and Quality Research Unit 468858 , Pullman, Washington, USA; The University of Tennessee Knoxville, Knoxville, Tennessee, USA

**Keywords:** Pseudomonas, wheat, *Brachypodium*, rhizosphere, water stress, root exudates, transcriptome

## Abstract

Arid and semi-arid regions, which account for over 40% of global land area, are affected by fluctuations in temperatures and rainfall. In these environments, plants recruit beneficial rhizosphere microorganisms to mitigate stress and enhance survival. This study investigates the molecular mechanisms by which *Pseudomonas synxantha* 2-79, a rhizobacterium associated with wheat grown in arid regions, adapts to water stress through its interaction with root exudates. We found that water-stressed wheat root exudates contain elevated levels of choline and glycine betaine, which serve as osmoprotectants for 2-79. Exposure to these exudates upregulated genes involved in the uptake and catabolism of these quaternary ammonium compounds (QACs), enhancing the bacterium’s ability to cope with osmotic stress. Mutants lacking QAC transporters displayed reduced growth under osmotic stress, highlighting the importance of these pathways in rhizosphere competence. Furthermore, the study revealed that 2-79 also produces biofilms containing protective exopolysaccharides, such as alginate and Psl, which aid in stress resilience. Overall, our findings provide insights into how root exudates shape bacterial adaptation to the water-stressed rhizosphere and highlight the role of QAC metabolism and biofilm formation in microbial survival and plant-microbe interactions under drought conditions.

**IMPORTANCE:**

This study advances our understanding of plant-microbe interactions in water-stressed environments by revealing how rhizobacteria adapt to osmotic stress through metabolic responses to plant-derived exudates. The utilization of compatible solutes such as choline and glycine betaine, which are abundant in water-stressed plants, contributes strongly to microbial survival and colonization of the dryland rhizosphere. By uncovering the molecular mechanisms underlying this adaptation, including the upregulation of QAC transporters and biofilm formation, the study highlights the potential to leverage beneficial microbes in sustainable agricultural practices. Understanding these interactions offers valuable insights for improving drought resilience in crops and developing microbiome-based strategies to enhance plant productivity in water-limited conditions.

## INTRODUCTION

Plants respond to water stress by employing a variety of morpho-physiological adaptations that result in stomatal closure, increased water uptake due to changes in the root architecture, and tissue-specific modulation of hormonal signaling to adjust osmotic processes (1). Plants also recruit beneficial rhizosphere microorganisms that positively influence plant fitness in response to stressors such as drought, salt, temperature, and soil pollution by modulating stress hormones, producing plant growth-promoting factors, maintaining the host’s nutritional status, and suppressing pathogenic stressors (2, 3). Recent studies have demonstrated that soil moisture shapes the belowground plant microbiome and that the drought-adapted rhizobiome confers water stress tolerance (4–8). It is generally accepted that the foundation for the differential affinity of rhizobacteria towards water-stressed host plants is built upon the interaction of microorganisms with complex secretions, lysates, and mucilages exuded by plant roots (9). However, in contrast to the well-studied mechanisms of drought tolerance in plants, the physiological and molecular events behind the recruitment of beneficial rhizobacteria and their adaptation to low water-content habitats remain poorly understood.

Microorganisms employ diverse physiological defensive mechanisms to cope with the deleterious effects of water stress. One type of defense response involves aggregation and formation of biofilms. Biofilm matrices contain various exopolysaccharides, extracellular DNA, and proteins that retain water and create a hydrated environment that protects bacteria from desiccation and other environmental stressors (10). Another widespread survival strategy involves the accumulation of neutral metabolites, referred to as compatible solutes, osmolytes, or osmoprotectants, which help maintain homeostasis in hyperosmotic environments such as arid or saline soils (11, 12). Common microbial osmoprotectants include certain sugars, polyols, amino acids, amino acid derivatives, and quaternary ammonium compounds (QACs) such as choline, glycine betaine, and carnitine (13). Bacteria can synthesize compatible solutes *de novo* or uptake them from the environment for osmoprotection or catabolism (14, 15). Compatible solutes not only aid in maintaining the osmolarity of bacterial cells, thereby resisting dehydration, but also provide resistance against temperature stress (16), thus increasing the ability of bacteria to proliferate in adverse ecological niches.

This study explored microbial responses to the water-stressed rhizosphere by focusing on molecular interactions between plants and *Pseudomonas synxantha* 2-79, a strain that exemplifies a defined group of beneficial rhizobacteria that are specifically associated with wheat grown in arid parts of the Pacific Northwest (17, 18). The molecular mechanisms involved in the enrichment of 2-79-like bacteria in dryland wheat are currently unknown. We have previously investigated genome-wide transcriptomic responses to root exudates in several well-characterized rhizosphere pseudomonads, including *P. synxantha* 2-79 (19). Our results revealed that exometabolites secreted by plant roots perturb numerous microbial genes encoding diverse enzymes, transporters, regulatory, stress response, and conserved hypothetical proteins. Notably, some of the differentially expressed genes were predicted to function in the uptake and catabolism of quaternary ammonium compounds, prompting us to investigate the contribution of these pathways to the adaptation of microorganisms to the water-stressed rhizosphere lifestyle. In this study, we generated root exudates from wheat, the primary host of *P. synxantha* 2-79, grown under water-replete and water-stressed conditions and performed their metabolomic profiling. We demonstrated that water-stressed root exudates contain elevated levels of choline and glycine betaine and protect 2-79 from osmotic stress. The exposure of 2-79 to these exudates was accompanied by the upregulation of QAC uptake and catabolism genes. We then inactivated genes involved in the uptake of QACs and tested the effect of these mutations on the ability of 2-79 to catabolize and use these metabolites as osmoprotectants. Finally, we used *Brachypodium distachyon* Bd21, a model species for economically important monocot plants (20), to determine the importance of QAC uptake genes for the rhizosphere competence of 2-79 under control and water-limiting conditions.

## MATERIALS AND METHODS

### Bacterial strains and culture conditions

*Pseudomonas synxantha* 2-79 and its mutants were routinely cultured at 27°C in Luria-Bertani (LB) medium (21) or King’s medium B (22). *Escherichia coli* DH5α was used in all gene cloning experiments and was grown in LB medium at 37°C. The RNA-seq and osmoprotection assays were performed by culturing 2-79 in 21C medium supplemented with 10 mM glucose or glucose and 20 mM pyruvate (14, 15). All strains were maintained as frozen glycerol stocks at −80°C. When appropriate, the growth media were amended with the following antibiotics: cycloheximide (100 µg mL^−1^), chloramphenicol (30 µg mL^−1^), rifampicin (100 µg mL^−1^), or gentamicin (15 or 90 µg mL^−1^).

### Extraction and metabolomic profiling of root exudates

Seeds of hard red spring wheat (cultivar Tara) were surface-sterilized with chlorine gas (23). The seeds were placed in open 50 mL conical centrifuge tubes inside an airtight glass desiccator, along with a beaker containing 100 mL of fresh commercial chlorine bleach mixed with 3 mL of concentrated HCl. The desiccator was sealed and placed in a fume hood for 16 h. The germination rate of sterilized seeds was monitored on moistened germination paper and ranged from 60% to 80% after 48 h at 20°C. To collect root exudates, sterile germinated seeds were placed into 1 L wide-mouth glass jars containing glass beads and distilled water. The jars were moved into an environmental chamber (Percival Scientific, Perry, IA), and seedlings were grown for 6 days at 18°C, with 600 µmol m^−2^ s^−1^ light intensity, using a 20-h light and 4-h dark cycle (19). Afterward, some of the plants were exposed to water stress by placing their roots in 0.3 M NaCl for 24 h, while the control plants were kept in distilled water. The stress level was selected based on previous studies that examined salinity tolerance responses in durum and bread wheat (24). Seedlings were then collected, and the roots were rinsed in several changes of sterile distilled water. They were then incubated in 25 mL of the same water for 3 h, after which exudates were collected, lyophilized, and shipped for metabolomic profiling to the Analytical Resources Core - Bioanalysis and Omics (ARC-BIO) at Colorado State University.

The samples were extracted with aqueous methanol and analyzed using gas or liquid chromatography coupled mass spectrometry. Briefly, samples were dissolved in ice-cold 80% methanol and spiked with several internal standards. The mixtures were vortexed and incubated for 3 h at −20°C, followed by centrifugation at 17,000 × *g* for 15 min at 4°C. The supernatants were dried under nitrogen and subjected to a two-step derivatization for GC-MS analysis. Metabolites were detected using a Trace 1310 GC coupled to a Thermo ISQ mass spectrometer (Thermo Fisher Scientific, Waltham, MA). Samples (1 µL) were injected into a 30 m TG-5MS column (0.25 mm i.d., 0.25 µm film thickness) (Thermo Scientific), with a 1.2 mL min^−1^ helium gas flow rate. The GC inlet was held at 285°C. The oven program started at 80°C for 30 s, followed by a ramp of 15°C min^−1^ to 330°C, and an 8-min hold. The split ratio was 10. Malic acid and fructose detections used different oven programs. The transfer line and ion source were held at 300 and 260°C, respectively. Masses between 50 and 650 m/z were scanned at five scans s^−1^ under electron impact ionization. The injector split ratio and oven temperature gradient varied with assays .

The LC-MS/MS analysis was performed on an Acquity Classic UPLC coupled to a Xevo TQ-S triple quadrupole mass spectrometer (both from Waters, Milford, MA). Chromatographic separations were carried out on an Acquity UPLC BEH Amide column (2.1 × 100 mm, 1.7 µM) (Waters) operated at 30°C. Mobile phases were (A) water with 10 mM ammonium formate and 0.1% formic acid and (B) acetonitrile with 0.1% formic acid. The following gradient was applied at a flow rate of 0.4 mL min^−1^: time = 0 min, 99% B; time = 0.5 min, 99% B; time = 7 min, 5% B; time = 11 min, 5% B; time = 11.5 min, 99% B; and time = 15 min, 99% B. Samples were held at 6°C in the autosampler, and the injection volume was 3 µL. The mass detector was operated in ESI mode with the capillary voltage set to 0.7 kV and inter-channel delay set to 3 msec. The source temperature was 150°C, and the desolvation gas (nitrogen) temperature was 450°C. Desolvation gas flow was 1000 L h^−1^, cone gas flow was 150 L h^−1^, and collision gas (argon) flow was 0.15 mL min^−1^. The nebulizer pressure (nitrogen) was set to 7 Bar, and the MS acquisition functions were scheduled by retention time. The autodwell feature was set for each function and dwell time was calculated by Masslynx software (Waters) to achieve >12 points across peak as the minimum data points per peak. Because of wide concentration ranges of analytes, each sample was injected three to four times with different dilutions.

GC-MS data were processed with Chromeleon 7.2.10 software (Thermo Fisher Scientific), while LC-MS/MS data files were imported into the Skyline package (25). Each target analyte was visually inspected for retention time and peak area integration. Peak areas were extracted for the target compounds detected in biological samples and normalized to the peak area of the appropriate internal standard or surrogate in each sample. Absolute quantitation was performed using linear regression equations obtained from calibration curves created for each compound.

### Profiling of 2-79 transcriptome responses to root exudates by RNA-seq

To profile transcriptome responses of strain 2-79 to wheat root exudates, bacteria were grown overnight on 21C-glucose plates, after which the cells were scraped off, washed, and suspended in a liquid 21C-glucose medium (26). The 10-fold concentrated solutions of root exudates were prepared by reconstituting the lyophilized material from water-replete and water-stressed plants in an appropriate volume of 21C medium. These concentrated root exudates were filter-sterilized and mixed at a 1:1 ratio with 2-79 cell suspension to achieve the density of 10^7^ CFU mL^−1^. Bacteria inoculated in 21C-glucose medium served as the control. The cultures were dispensed in 96-well microtiter plates and incubated statically at 27°C till mid-exponential phase (OD_630_ of 0.5-0.6), after which the bacteria were harvested, stabilized in RNAprotect reagent (QIAGEN, Germantown, MD), and extracted with a RNeasy Protect Bacteria Mini Kit (QIAGEN). The extracted RNA was treated with TURBO DNase (Thermo Fisher Scientific) and quantified using the QuantiFluor RNA system (Promega, Madison, WI). The RNA integrity was verified using a 2100 Bioanalyzer and an RNA 6000 Nano Kit (both from Agilent Technologies, Santa Clara, CA), and all samples were shipped to Novogene (Sacramento, CA, USA). Four biological replicates of RNA per condition were ribodepleted with Ribo-Zero Plus rRNA Depletion Kit (Illumina, San Diego, CA), and stranded RNA-seq libraries were prepared and sequenced in PE150 mode on a NovaSeq 6000 instrument (Illumina).

The sequence reads were imported in KBase (27), quality filtered with FastQC v.0.11.5 (https://www.bioinformatics.babraham.ac.uk) and aligned with HISAT2 v.2.1.0 (28) to the complete 2-79 genome (GenBank accession number CP027755). Full-length transcripts were assembled with StringTie v.13.3 (29), and differential expression analysis was carried out using DeSeq2 v.1.20.0 (30). Genes differentially expressed (i.e., absolute fold change >1 (log_2_ scale), FDR-adjusted *P*-value (*q*-value) <0.01) between the control and experimental treatments were used for downstream analysis. The functional categorization of differentially expressed genes and gene enrichment analyses was performed using Blast2GO annotation (31) in OmicsBox (BioBam Bioinformatics, Cambridge, MA). The gene ontology (GO) terms were derived from 50 different functional groups from GO subcategory levels 3 and 4. The taxonomic distribution of selected metabolic pathways was analyzed with AnnoTree (32) using the following cutoff parameters: % identity >30, *e*-value <0.00001, % subject alignment >70, and % query alignment >70.

### Reverse transcription-quantitative PCR assays

Extracted RNA was converted to cDNA with a First-Strand cDNA Synthesis Kit (New England Biolabs, Ipswich, MA) and analyzed in RT-qPCR assays performed with the Luna Universal qPCR Master Mix (New England Biolabs) and primer sets targeting the QAC catabolism genes *gbcA*, *dgcA*, and *soxB* (Table S1). The expression of target genes was normalized to that of the housekeeping gene *rpoD* amplified with primers rpoDf and rpoDr. The assay was performed using a CFX96 Real-Time PCR Detection System and CFX Maestro software (Bio-Rad Laboratories, Hercules, CA).

### Construction of isogenic mutants

Selected *P. synxantha* 2-79 pathways involved in the uptake and synthesis of osmoprotectants and production of biofilm matrices were subjected to allelic gene replacement mutagenesis. Briefly, genome regions flanking each target gene were amplified by PCR using Q5 Hot Start High-Fidelity Master Mix (New England Biolabs) or Phusion High-Fidelity DNA polymerase (Thermo Fisher Scientific), with primers listed in Table S1. The amplicons were processed with a GeneJet PCR Purification Kit (Thermo Fisher Scientific) or, if necessary, separated on a preparative gel and extracted from agarose with a QIAEX II Gel Extraction Kit (QIAGEN). The gene replacement vector pEX18Gm (33) was linearized by inverse PCR with primers rM13f and rM13r. The purified amplicons were ligated with pEX18Gm using isothermal assembly with the NEBuilder HiFi Master Mix (New England Biolabs) and transformed into *E. coli* DH5α competent cells. The transformants were selected on LB-gentamicin agar and screened for the presence of cloned amplicons by colony PCR with M13f and M13r primers (Table S1). The confirmed recombinant plasmids were purified using a GeneJet Plasmid Miniprep Kit (Thermo Fisher Scientific) and verified by sequencing at Eurofins Genomics (Louisville, KY). The confirmed plasmids were electroporated into *P. synxantha* 2-79 Rif, a spontaneous rifampicin-resistant mutant of 2-79, and single crossover mutants were selected by plating on LB amended with 90 µg mL^−1^ gentamicin. To select double crossovers, several gentamicin-resistant clones were inoculated in 0.5 mL LB broth, grown for 2–3 h at 27°C with shaking at 220 rpm, and plated on LB supplemented with 5% sucrose. The resultant mutants were screened for the presence of desired deletions by PCR with appropriate gene-specific forward primers and the deletion-specific primer Stop (Table S1). The PCR was also done with *sacB-* and *aacC1-*specific primer sets to confirm the loss of the pEX18Gm backbone.

### Growth on QAC compounds

The ability to catabolize quaternary ammonium compounds (QACs) was tested by growing 2-79 overnight on full-strength 21C agar amended with 10 mM glucose and 20 mM pyruvate. The cells were scraped, washed, and inoculated at 10^6^ CFU mL^−1^ in liquid half-strength 21C medium amended with 20 mM QAC carbon source (choline, glycine-betaine, sarcosine, or carnitine). The cultures were dispensed into 96-well microtiter plates and incubated at 27°C for 48 h. The bacterial growth was monitored by taking hourly measurements of optical density at 600 nm with a Synergy 2 microplate reader (BioTek, Winooski, VT).

### Osmoprotection assays

The bacteria were cultured overnight on half-strength 21C glucose-pyruvate agar, after which the cells were collected, washed, and suspended in the same liquid medium at 10^6^ CFU mL^−1^. The osmotic or matric stress was imposed through the addition of varying concentrations of sodium chloride (NaCl) or polyethylene glycol 8000 (PEG). To test the ability of QACs to rescue the growth of these water-stressed cultures, the growth medium was also supplemented with 1 mM choline, glycine betaine, sarcosine, carnitine, or 10-fold concentrated root exudates dissolved in half-strength 21C medium. All cultures were incubated statically in 96-well microtiter plates for 48 h at 27°C, and the growth was monitored by measuring absorbance at 600 nm. The OD_600_ readings were converted to an area under the growth progress curve (AUGPC) using the trapezoidal integration method (34), and differences between treatments were analyzed by ANOVA or Kruskal–Wallis test (*P* < 0.05).

### Ice nucleation activity (INA) reporter assays

To characterize the impact of water stress on strain 2-79, promoters of selected water stress response genes were fused to the ice nucleation reporter gene *inaZ* in the stable broad-host-range vector pPROBE-TI (35). Briefly, to construct the *betT1* reporter, the target promoter region of this gene was amplified by PCR with Phusion DNA polymerase (Thermo Fisher Scientific) and primers betT1F/betT2RinaZ (Table S1). The amplicon was purified with GeneJet PCR Purification Kit (Thermo Fisher Scientific) and cloned into pPROBE-TI digested with *Eco*RI and *Hin*dIII using the Gibson Assembly Master Mix (New England Biolabs). Reporter plasmids containing the promoters of *cbcX* and *opuC* were constructed identically using primer sets cbcXF/cbcXRinaZ and opuCF/opuCRinaZ, respectively (Table S1). The resultant plasmids were verified by sequencing and electroporated into *P. synxantha* 2-79. Clones carrying the *inaZ* reporter plasmids were selected and maintained on LB medium amended with 25 µg mL^−1^ tetracycline.

The *in planta* expression of the *inaZ* reporter in derivatives of 2-79 was measured as follows. Seeds of *B. distachyon* Bd21 were surface-sterilized with 1.5% NaOCl, rinsed twice with distilled water, stratified for three days at 4°C in the dark, and pregerminated for two days at room temperature on 0.8% water agar. The pregerminated seeds were inoculated by soaking for 30 min in water containing 10^7^ CFU mL^−1^ of an appropriate bioreporter strain. The control plants were treated only with water. The bacterized seeds were placed in CYG seed germination pouches (Mega International, Newport, MN) moistened with sterile 0.2 × Hoagland’s solution (36). The pouches with plants were incubated for five days in an environmental chamber (Percival Scientific, Perry, IA) under 20-h light (150 µmol m^−2^ s^−1^), 24°C/4-h dark, and 18°C cycle. On the fifth day, the plants were water-stressed by adding to each pouch 6 mL of 0.1 M NaCl (osmotic water stress) or 10% PEG (matric water stress) and incubated in the environmental chamber for two additional days. The control treatments were treated with sterile water.

To measure the INA activity, five or six root systems were placed into a 50 mL centrifuge tube containing 10 mL sterile distilled water. Each centrifuge tube was treated as a replicate, and each bioreporter was assayed in triplicate. The tubes were vortexed and treated for 1 min in an ultrasonic bath (Thermo Fisher Scientific). The resultant root wash was used to enumerate bacteria by the microplate endpoint dilution assay (18), while the ice nucleation activity (INA) was measured by the droplet freezing assay (37). Serial 10-fold dilutions were prepared in phosphate-buffered saline (pH 7.0) solution, and forty 10 µL droplets from each dilution were pipetted onto the surface of a foil boat placed in a supercooled (−5°C) circulating bath. After 2 min, frozen droplets were counted, and dilutions containing four to 34 frozen droplets were selected to determine the level of active ice nuclei. INA values were calculated by normalizing the number of active ice nuclei to the number of bacterial cells as follows: INA = $\frac{icenuclei/mL}{CFU/mL}$ . Ice nuclei per milliliter was calculated using the following formula: ice nuclei / mL = $\frac{\ln\left(\frac{N_{o}}{N_{o}-N_{f}}\right)}{V}\times \frac{1}{D}$ , where *N_o_* is the total number of droplets tested; *N_f_* is the number of droplets that froze; *V* is the volume of each droplet; and *D* is the dilution from the original culture that was used in the assay.

### Rhizosphere fitness assays

The rhizosphere fitness assays were performed with *B. distachyon* Bd21 in an environment chamber (Percival Scientific) under 20-h light (150 µmol m^−2^ s^−1^), 24°C/4-h dark, and 18°C cycle. The Bd21 seeds were surface-sterilized (19), stratified, and planted into 8.9 × 6.5 cm plastic pots filled with Sunshine Potting Mix #4 (SunGro Horticulture, Agawam, MA). Plants were water-stressed by maintaining the desired relative soil water content (RSWC) as described by Bertolini et al. (38). The plant assay lasted 3 weeks, and during the first week, all pots were watered to 80% RSWC and fertilized once with the Peters Professional 20-20-20 General Purpose fertilizer (ICL Specialty Fertilizers, Summerville, SC). After seven days, the control pots were kept at 80% RSWC, while the rest of the plants were allowed to dry down to 40% RSWC to impose moderate drought stress. The target moisture level was then maintained by weighing and watering pots daily 8 h after the onset of the light. The location of pots for each treatment was alternated every other day to minimize the differential effect of light intensity on plant growth. Each treatment was replicated six times, with one pot serving as a replicate, and the experiment was conducted twice.

The effect of water stress was evaluated by measuring the shoot length and leaf relative water content (RWC). For the RWC measurements, 4–5 cm long segments of fully expanded leaves were taken from control and water-stressed plants, covered in plastic wrap to eliminate evaporation, and stored in the dark before measuring fresh weight (FW). For measuring turgid weight (TW), the leaves were hydrated in distilled water for 5 h at room temperature in the dark, after which they were dried briefly with filter paper and weighed. Dry weight (DW) was obtained after oven-drying the leaf samples at 80°C for 24 h. RWC was determined as follows: RWC = (FW − DW)/(TW − DW) × 100 (39).

*Brachypodium* was inoculated with the wild-type *P. synxantha* 2-79 and its isogenic mutants by suspending freshly grown bacteria in phosphate-buffered saline (pH 7.5) in 1% methylcellulose and incorporating them at 10^5^ CFU g^−1^ into potting mix before planting seeds. The rhizosphere fitness of mutants was tested individually and in competition with the wild-type 2-79 by co-inoculating plants with two strains at a 1:1 ratio. The inoculated plants were grown in control (80% RSWC) and water-stressed (40% RSWC) conditions for three successive 3-week cycles. After each cycle, plants were removed from the pots, and their root systems were excised and placed in 50 mL centrifuge tubes containing 10 mL of sterile distilled water. The tubes were vortexed for 1 min and treated for 1 min in a sonicating water bath to dislodge the bacteria from plant roots. Bacterial population densities in the resultant root wash were determined using the microplate terminal dilution assay in 1/3-strength King’s medium B with cycloheximide and rifampicin, as described by Mavrodi et al. (40, 41). The inoculated microplates were incubated at room temperature for 72 h, and wells with OD_600_ >0.1 were considered positive for bacterial growth. The mean population density data were converted to log CFU g root^−1^ and statistically analyzed by ANOVA or Kruskal–Wallis test (*P* < 0.05). Population levels of mutant strains in mixed inoculations were determined by screening the microplate wells by PCR with an appropriate gene-specific forward primer and the reverse stop primer (Table S1). The amplifications were performed with DreamTaq DNA polymerase (Thermo Fisher Scientific).

## RESULTS

### Water stress significantly alters the composition of wheat root exudates

**Fig 1:**
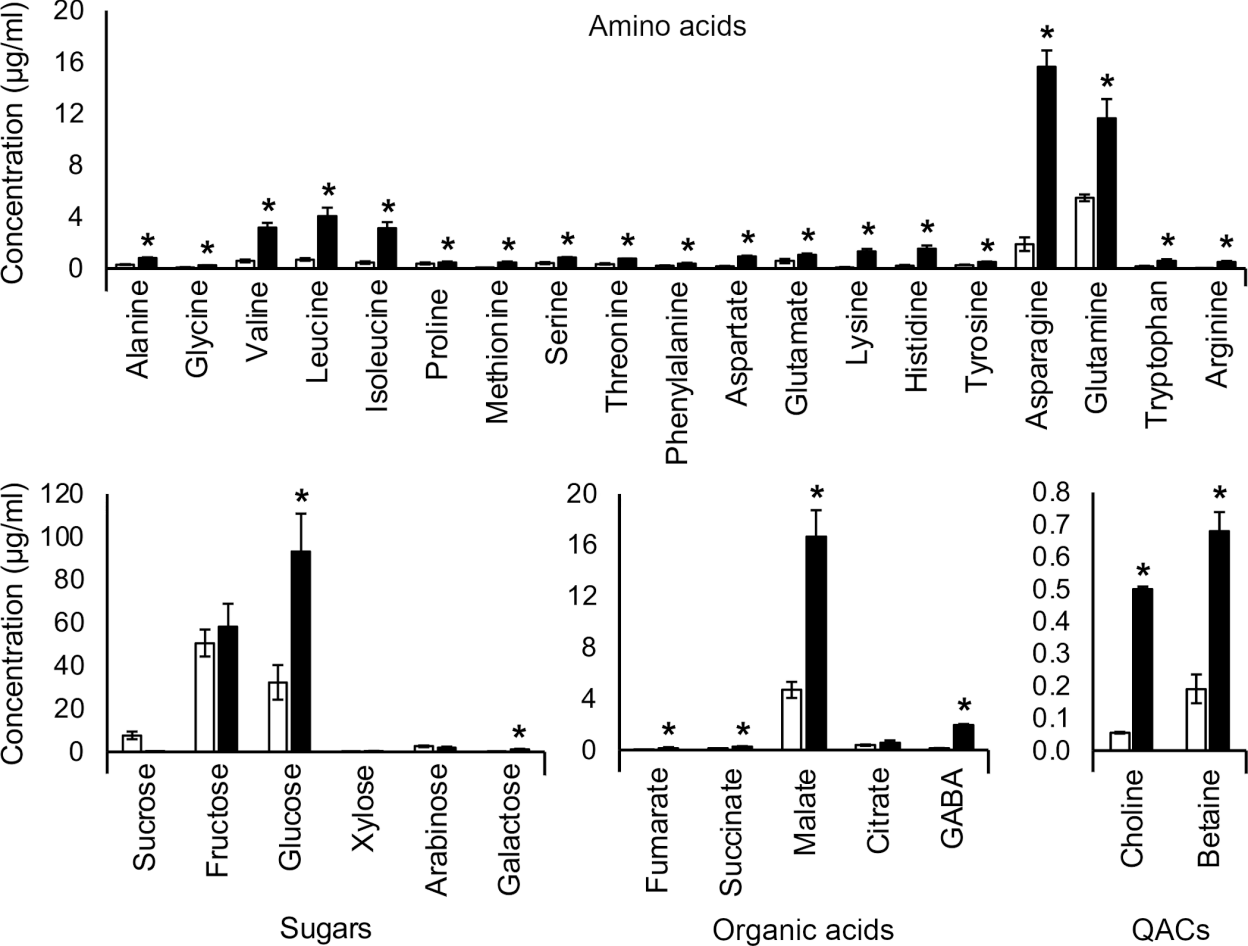
Concentrations (mean ± SD) of primary metabolites and quaternary ammonium compounds (QACs) in water-replete (white bars) and water-stressed (black bars) exudates of spring wheat cv. Tara. Asterisks indicate significant differences in metabolite concentrations, as assessed by Student’s *t*-test (*P* < 0.05).

To evaluate the effect of water stress on rhizodeposition, we generated root exudates from water-replete control and osmotically stressed spring wheat cv. Tara plants and compared their composition by metabolomic profiling. The analysis demonstrated the presence of diverse amino acids, carbohydrates, and organic acids (Fig. 1). Both types of exudates contained all common amino acids except cysteine, with particularly high levels of asparagine and glutamine. The exudates also contained significant levels of QACs, especially choline and glycine betaine (GB). We also identified carnitine, which coeluted with interfering substances, as well as sarcosine, which coeluted with alanine and hence was not detected separately. The quantitative analysis revealed that water stress significantly alters the levels of multiple metabolites. The water-stressed plants secreted elevated amounts of amino acids (especially asparagine, glutamine, leucine, isoleucine, and valine), glucose, malate, succinate, fumarate, and γ-aminobutyric acid (GABA). Most notably, the stress also resulted in significantly higher concentrations of choline and glycine betaine, which function as key osmoprotectants in plants and diverse groups of microorganisms.

### Transcriptome responses of 2-79 to root exudates of water-replete and water-stressed plants

**Fig 2:**
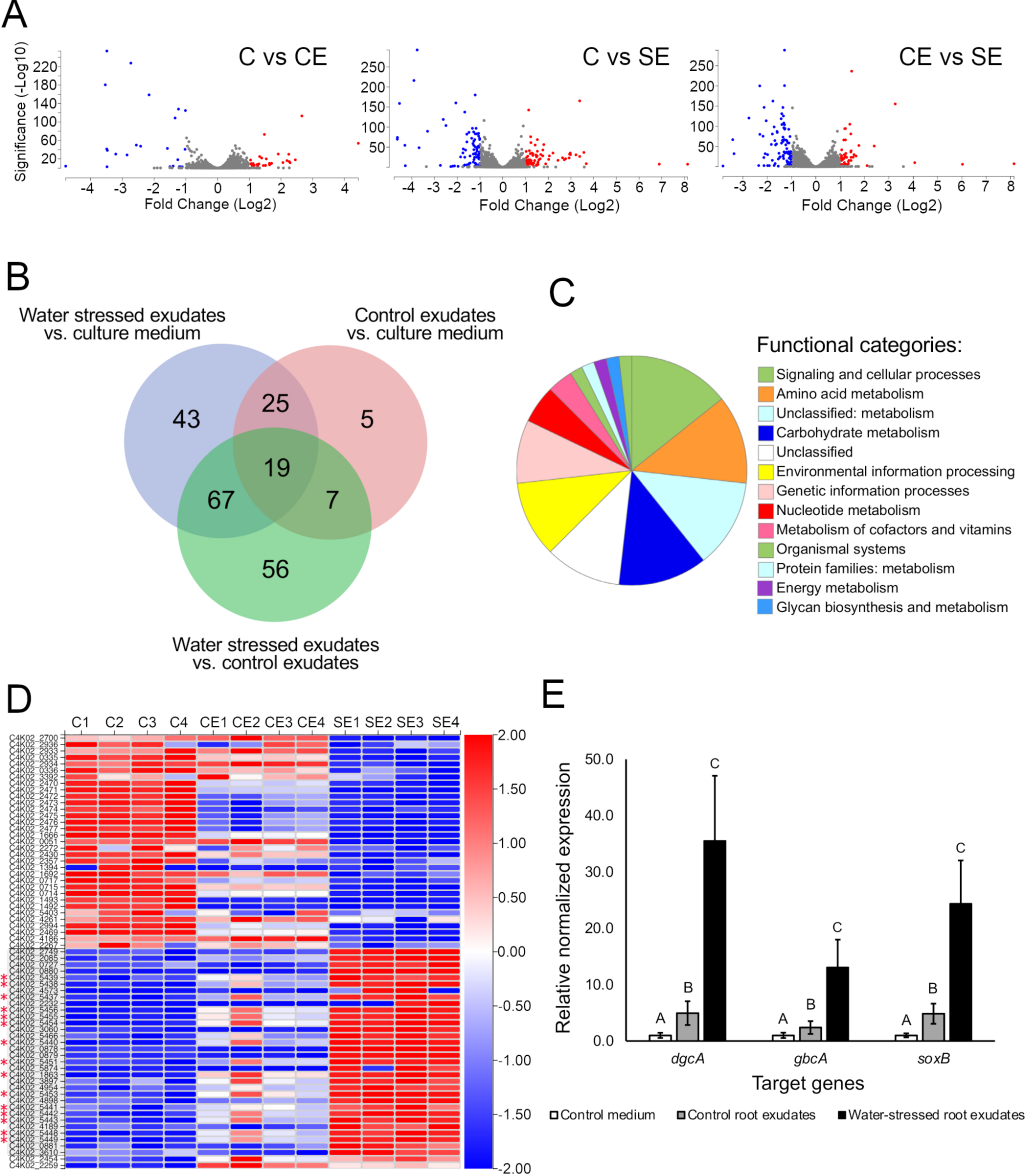
Transcriptome responses of 2-79 to wheat root exudates. (**A**) Volcano plots showing significantly upregulated and downregulated genes in red and blue (C, culture medium; CE, control exudates; SE, water-stressed exudates). (**B**) Venn diagram of differentially expressed genes (DEGs) that are unique and shared between treatments. (**C**) KEGG Orthology classification of proteins encoded by DEGs. (**D**) Changes in the expression of selected DEGs. Red asterisks indicate genes involved in QAC transport and metabolism. (**E**) Relative expression of key QAC catabolism genes in response to the culture medium, water-replete, and water-stressed root exudates, as measured by RT-qPCR. Different letters indicate significant differences in gene expression, as analyzed by ANOVA followed by Tukey-Kramer’s HSD test (*P* < 0.05).

The RNA-seq experiment, with bacteria grown in the control conditions and in the presence of wheat water-replete and water-stressed root exudates, generated a total of 235.3 million high-quality Illumina sequencing reads (~19.6 million reads per sample) that were aligned onto the 2-79 genome. Statistical analysis using a *q*-value cutoff of 0.01 and an absolute log_2_ fold change threshold of 1.0 revealed a total of 166 genes that were differentially expressed in response to wheat root exudates. Of these, 56 and 43 genes were differentially expressed, respectively, in response to water-replete and water-stressed root exudates (Fig. 2B; Table S2). The KEGG Orthology (KO) classification revealed that most of the differentially expressed genes (DEGs) are predicted to function in signaling and cellular processes, amino acid metabolism, carbohydrate metabolism, processing of environmental and genetic information, and metabolism of nucleotides and cofactors (Fig. 2C).

Genes upregulated by water-replete exudates are predicted to function in the uptake and catabolism of *myo*-inositol (42) and *D*-xylose (43), as well as in the transport of GABA, tricarboxylic acids, and 4-hydroxybenzoic acid. Stress response genes were also induced, including those encoding an organic hydroperoxide resistance protein, the pyoverdin-specific sigma factor PvdS, and DksA, a regulator of virulence, metabolic adaptation, and survival via interactions with RNA polymerase and ppGpp (44). Downregulated genes encoded conserved hypothetical proteins and those involved in metal ion transport, multidrug efflux, and prophage functions.

**Fig 3:**
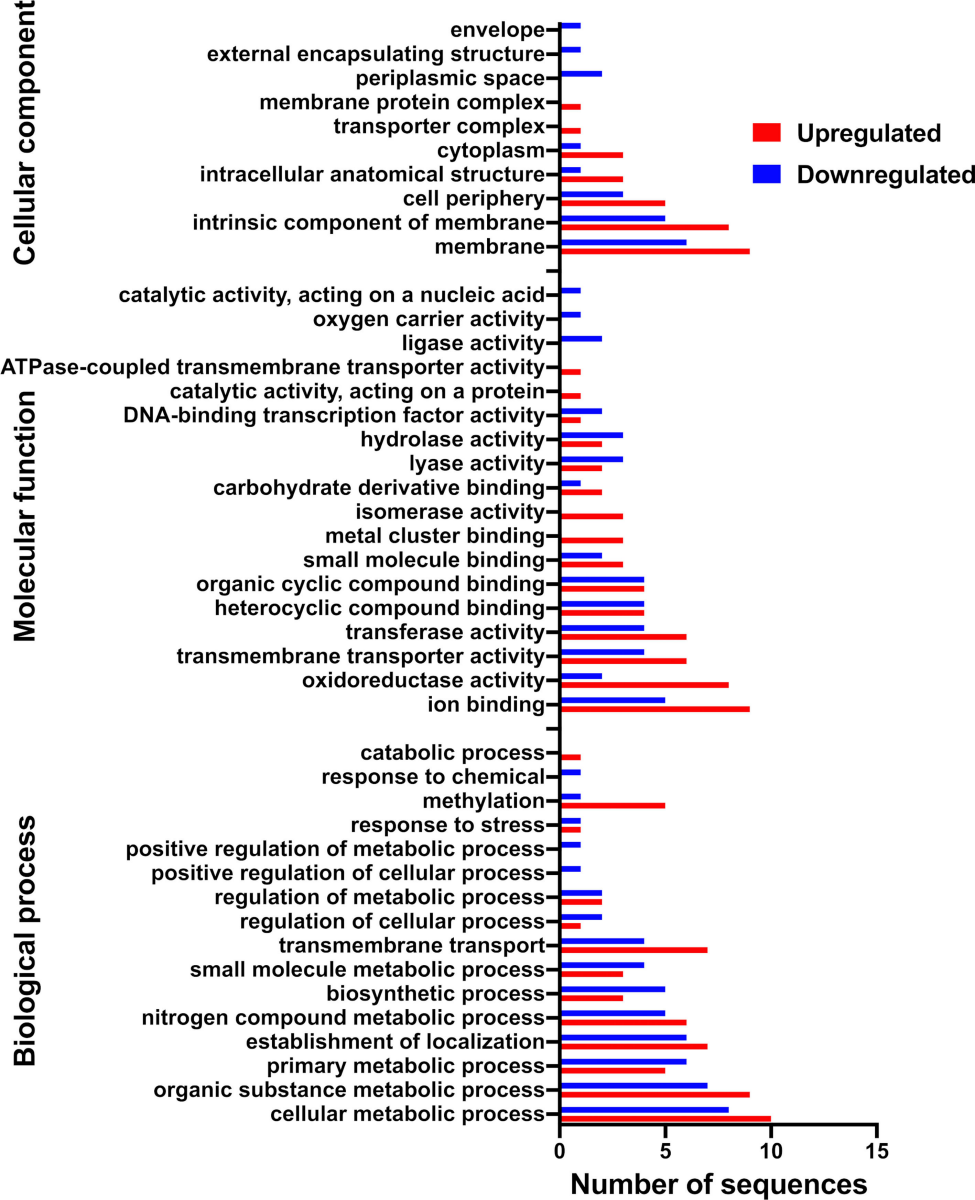
Gene ontology (GO) annotation of 2-79 genes that are differentially expressed in response to water-replete wheat root exudates. The GO terms were derived from 50 distinct functional groups (GO subcategory level 3).

Gene ontology (GO) analysis identified membrane, its intrinsic components, and transporter and membrane protein complexes as key cellular components induced by water-replete exudates (Fig. 3). At the molecular function level, upregulated genes were linked to activities such as ions and metal cluster binding, oxidoreductases, transmembrane transporters, transferases, isomerases, and electron transfer. Upregulated biological processes included cellular and organic substance metabolism, transmembrane transport, and methylation. Repressed genes were associated with the cell envelope, outer membrane, periplasmic space, and molecular functions such as lyase, hydrolase, ligase, and oxygen carrier activity. The repressed biological processes involved biosynthesis, small molecule metabolism, positive regulation of cellular and metabolic processes, and chemical response. GO term enrichment revealed an overrepresentation of genes involved in the metabolism of pteridine-containing compounds (GO ID 0042558) in response to water-replete exudates (Fisher’s exact test, FDR-adjusted *P*-value < 0.05).

 Treatment with water-stressed exudates upregulated genes involved in the uptake and catabolism of fructose, amino acids, and several hypothetical and regulatory proteins, including an extracytoplasmic function (ECF) sigma factor (Table S2). Downregulated genes were linked to the transport and catabolism of taurine, transport of ribose, efflux of heavy metals and antimicrobials, and prophage elements. Other important repressed genes included the redox-sensitive transcriptional activator SoxR and several proteins (e.g., ClpB, Lon, and HslUV protease components) linked to cellular homeostasis and protein turnover.

**Fig 4:**
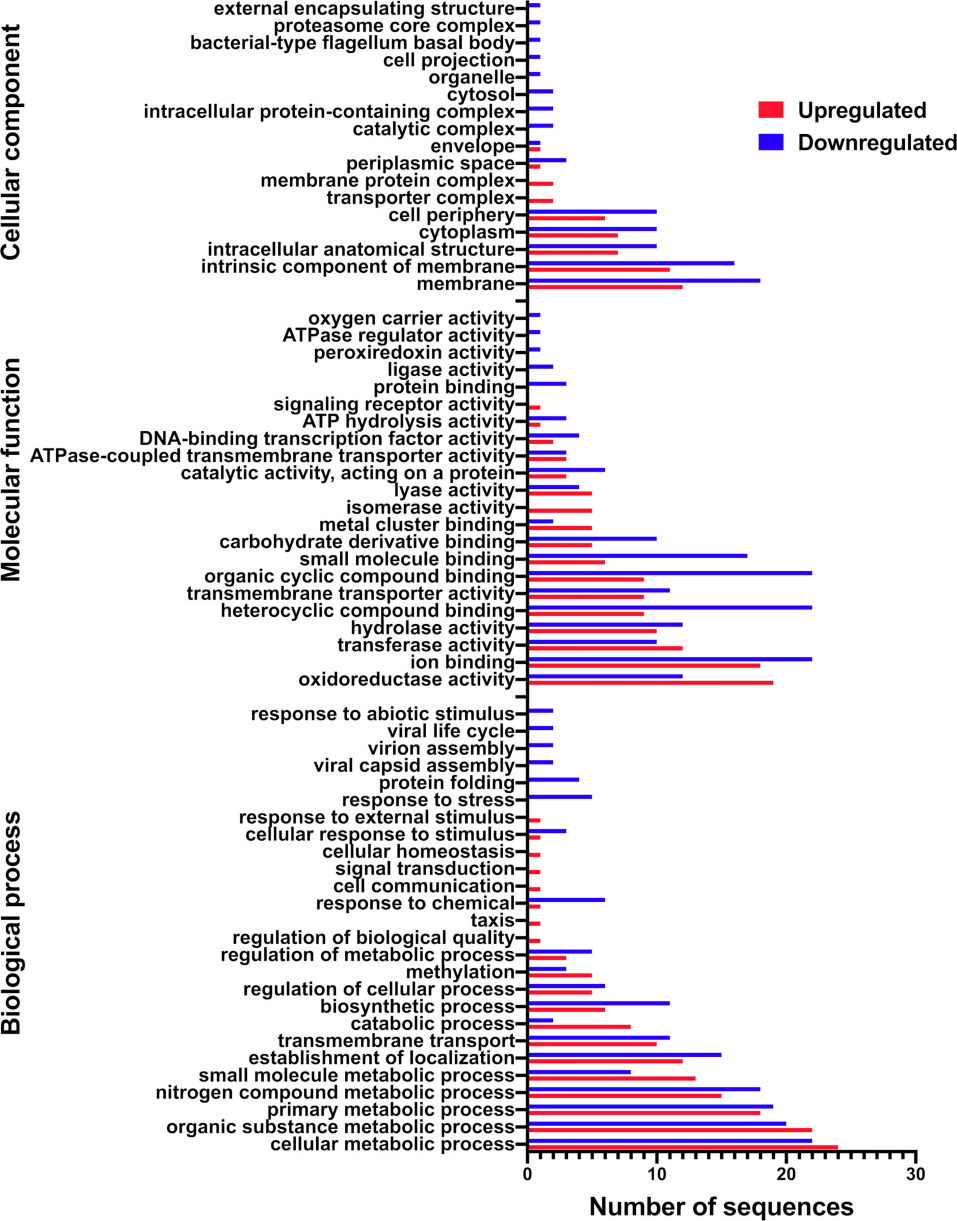
Gene ontology (GO) annotation of 2-79 genes that are differentially expressed in response to the root exudates of water-stressed wheat. The GO terms were derived from 50 distinct functional groups (GO subcategory level 3).

Genes upregulated by water-stressed exudates were linked to cellular components such as membrane proteins and transporter complexes (Fig. 4). Major molecular functions included oxidoreductase, transferase, metal cluster binding, and isomerase activities, while biological processes involved the metabolism of small molecules, catabolism, and methylation. Downregulated genes were associated with the membrane and its components, flagellum, and proteasome complexes, with functions like binding of ions, heterocyclic and organic cyclic compounds, small molecules, carbohydrates, and proteins. Repressed biological processes involved nitrogen metabolism, biosynthesis, response to chemicals and stress, protein folding, and viral functions. Enrichment analysis showed overrepresentation of genes with oxidoreductase (GO:0016645, GO:0016647) and sarcosine oxidase activity (GO:0008115) in response to water-stressed exudates (Fisher’s exact test, FDR < 0.05).

Interestingly, *P. synxantha* 2-79 also responded to root exudates by upregulating genes that encode the glycine betaine/proline transport system substrate-binding protein BetX (gene tag C4K02_1863); enzymes converting glycine betaine to dimethyl glycine (C4K02_5448 and C4K02_5449); those functioning to convert dimethyl glycine to sarcosine (C4K02_5451 through C4K02_5456); and those converting sarcosine to glycine (C4K02_5437 through C4K02_5443) (Table S2). Most of these genes were significantly upregulated in the presence of water-stressed exudates, agreeing with elevated levels of CHO and GB detected by the metabolome analysis. qPCR-based analysis of cultures exposed to water-stressed exudates confirmed significantly higher expression of genes encoding the key QAC catabolism enzymes GbcA (GB demethylase), DgcA (dimethylglycine demethylase), and SoxB (sarcosine oxidase) compared to cultures grown in the half-strength 21C medium with water-replete root exudates or glucose (Fig. 2D and E).

### QACs and root exudates protect 2-79 from water stress

**Fig 5:**
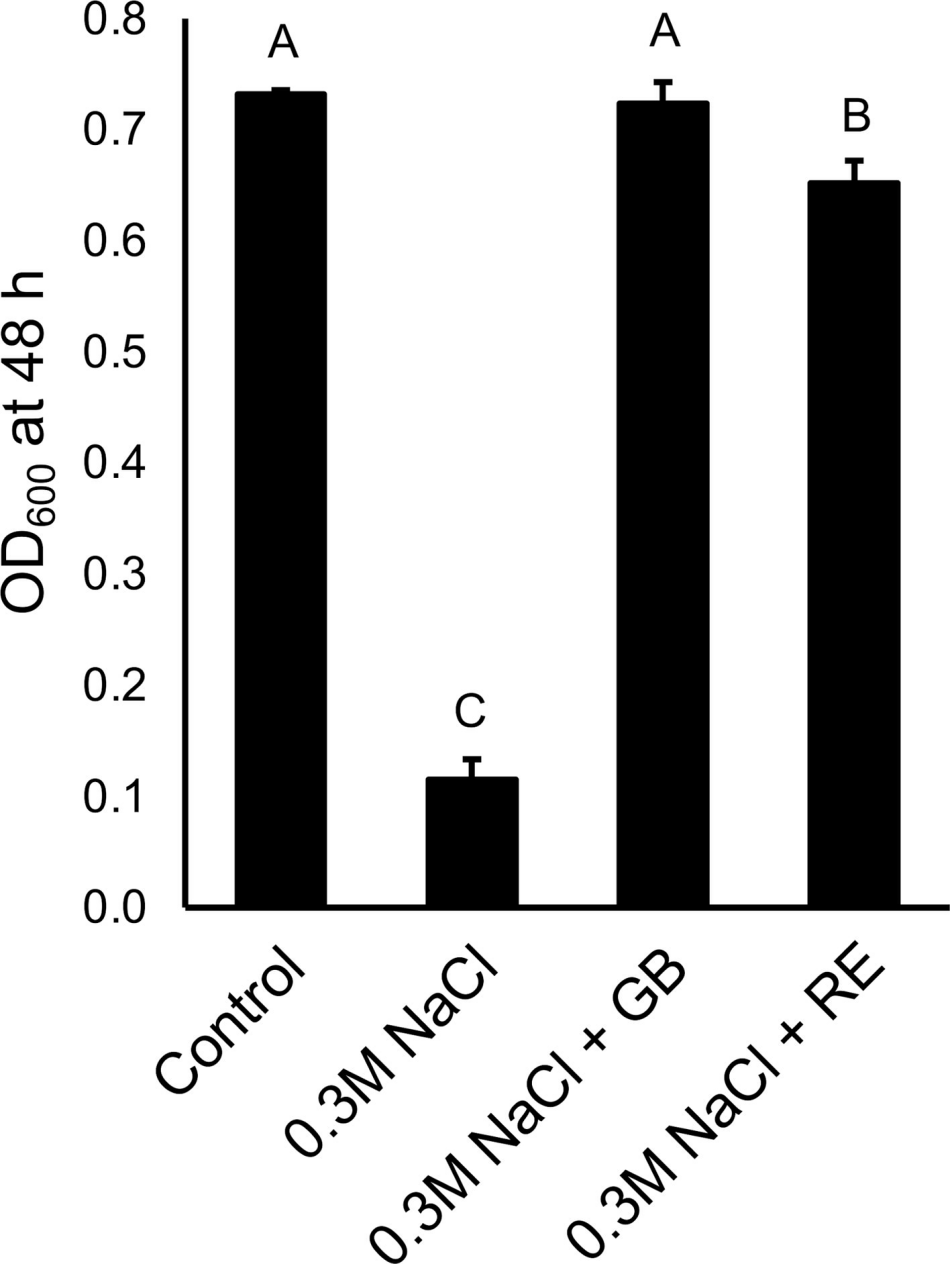
Root exudates from water-stressed wheat cv. Tara protect 2-79 against osmotic stress. The bacteria were cultured for 48 h in low-osmoticum half-strength 21C-glucose-pyruvate medium, and osmotic stress was imposed using 0.3 M NaCl. The growth medium was supplemented with 1 mM glycine betaine (GB) or 10 times concentrated root exudates (RE). Error bars represent standard deviation, and different letters indicate treatments significantly different from the control based on the Wilcoxon signed-rank test (*P* < 0.05).

To assess the effect of root exudates on the ability of *P. synxantha* 2-79 to cope with water stress, the strain was initially grown in half-strength 21C medium supplemented with varying concentrations of sodium chloride (NaCl) or polyethylene glycol 8000 (PEG). The assay revealed significant growth inhibition in the presence of >0.2 M NaCl or >10% w/v PEG 8000 compared to untreated controls. Therefore, subsequent water stress assays were performed using 0.3 M NaCl or 10–20% w/v PEG 8000. To investigate the protective effect of QAC osmoprotectants, *P. synxantha* 2-79 was cultured in half-strength 21C-NaCl medium with or without 1 mM choline, GB, sarcosine, or carnitine. After 48 hours, the control cultures were strongly inhibited, whereas cultures treated with choline, GB, and carnitine (but not sarcosine) demonstrated significantly better growth, indicating that these QACs acted as effective osmoprotectants in 2-79 (Fig. S1). These compounds improved bacterial growth under osmotic stress imposed by 0.2–0.5 M NaCl, but not under matric stress imposed by PEG 8000 (data not shown). In addition, the tested QACs did not provide protection from thermal stress and failed to improve the growth of 2-79 at low and elevated temperatures (data not shown). Since wheat rhizodeposits contained significant levels of choline and glycine betaine, we tested water-stressed root exudates for the ability to rescue bacterial growth under water stress. The root exudates also provided a significant protective effect similar to that of GB, restoring bacterial growth in NaCl-stressed cultures almost to control levels (Fig. 5).

### The genome of *P. synxantha* 2-79 encodes multiple classes of water stress response pathways

The genome sequence of 2-79 was compared to genomes of *P. aeruginosa*, *P. syringae*, and several well-studied strains of the *P. fluorescens* complex to analyze the presence and organization of water stress response pathways. The results revealed that 2-79 and closely related rhizosphere strains share a set of conserved pathways involved in: (i) the aggregation and formation of biofilms (exopolysaccharides, fimbriae, pili, and large adhesion proteins), (ii) the *de novo* synthesis of microbial osmoprotectants (trehalose and *N*-acetyl-glutaminylglutamine amide [NAGGN]), and (iii) the uptake and catabolism of quaternary amine (QA) osmoprotectants (Table S3).

Bacterial biofilms are complex cell communities embedded in an extracellular matrix of proteins, DNA, and exopolysaccharides (EPSs). *Pseudomonas synxantha* 2-79 produces two main EPSs: alginate and Psl. Alginate, a high-molecular-weight polymer of L-guluronic and D-mannuronic acids linked by β−1,4 bonds, is a conserved capsular polysaccharide across *Pseudomonas* species (10). In 2-79, 12 *alg* biosynthesis genes form a single operon, likely regulated by the *algU/T-mucABD* operon and the AlgZR two-component system, similar to *P. aeruginosa* (45, 46). Psl, composed of repeating units of D-mannose, L-rhamnose, and D-glucose, is encoded by an 11-gene *psl* locus (10). While common in the *P. fluorescens* group, complete *psl* clusters were found in only three of seven strains analyzed (Table S3). Another wheat isolate, R1-43-08, contains a partial *psl* locus, and its ability to produce Psl remains uncertain. Additionally, 2-79 possesses a unique 15-gene cluster related to polysaccharide biosynthesis, which is present in a few *P. fluorescens* strains but absent from *P. aeruginosa*, *P. syringae*, and *P. putida*. The presence of an O-antigen ligase suggests that these *eps* genes may modify O-antigen rather than synthesize an aggregative EPS.

The *P. synxantha* 2-79 genome encodes two trehalose biosynthesis pathways. The first involves *treY* and *treZ*, which convert glucan chains into trehalose via α- (1–4) to α,α- (1) linkage modification (47). The second uses *treS*, which reversibly converts maltose to trehalose and is co-transcribed with *glgE* and *glgB*, key genes in glycogen biosynthesis. Both TreYZ and TreS pathways are conserved across the *P. fluorescens* group. 2-79 also likely synthesizes the osmoprotectant N-acetyl glutaminyl glutamine amide (NAGGN) via *ggnA* and *ggnB*, encoding an amidotransferase and an N-acetyltransferase, respectively. These resemble enzymes from *P. syringae* and *Ensifer meliloti* (48, 49). While the canonical *ectABCD* genes for L-ectoine/5-hydroxyectoine biosynthesis are absent (50), 2-79 carries a single *ectC* gene, which is also found in *P. syringae* B728a and *P. fluorescens* SBW25.

Finally, *P. synxantha* 2-79 has multiple pathways for scavenging quaternary amines (QAs), such as choline, glycine betaine (GB), carnitine, choline-O-sulfate (COS), and sarcosine. QAs are imported through two types of transporters: ABC transporters (CbcWV and OpuC) and BCCT (betaine-carnitine-choline) family proton symporters (BetT1-3) (51, 52) (Table S3). Based on studies in *P. syringae* and *P. aeruginosa*, CbcWV likely functions under water-replete conditions with various substrate-binding proteins (SBPs) expected to bind choline (CbcX), GB (BetX), COS (CosX), and carnitine (CdhX). OpuC, encoded by *opuABCD*, is predicted to transport GB and choline in high osmolarity environments (14). Regulatory genes *betI*, *cdhR*, *cosR*, and *souR* were also identified, often next to relevant transport or catabolic operons (Fig. S2). A conserved *betI* binding site was found upstream of *betT1*, similar to sites in *P. aeruginosa*, *E. coli*, and *Ensifer meliloti* (Fig. S3). Additionally, the AraC-family regulator *gbdR* is present and predicted to control 15 genes in 2-79, including those involved in QA transport/catabolism and detoxification of byproducts (53), as well as MFS transporters, a GABA permease, and hypothetical proteins.

To evaluate the role of QAC and biofilm pathways in water stress resistance, we generated isogenic mutants of 2-79 targeting genes for alginate, Psl, and Eps production, as well as *ggnA* and *treS*, which are involved in NAGGN and trehalose synthesis. We also disabled genes encoding the BCCT transporters BetT1–3, components of the ABC transporters Opu and Cbc, and the solute-binding protein CbcX. Under NaCl-induced osmotic stress, only mutants lacking NAGGN or trehalose synthesis showed significantly reduced growth, while other mutants appeared similar to the wild type (Fig. S4).

### *P. synxantha* 2-79 utilizes QACs both as nutrients and osmoprotectants

We further compared the ability of transporter mutants to catabolize quaternary ammonium compounds and use them as osmoprotectants. When QACs were supplied as a sole carbon source, the wild-type 2-79 utilized choline, GB, and sarcosine (but not carnitine) for growth both under water-replete conditions and under osmotic stress (Table 1). The best growth was observed in the medium amended with GB. In contrast, the CbcXWV mutant lost the ability to catabolize choline, while the double BetT1/BetT2 knockout grew slightly slower on this carbon source. Interestingly, the rest of the mutants, including the multiple Δ*betT1*/Δ*betT2/ΔbetT3*/Δ*cbcXWV*/Δ*opuC* gene knockout, retained the ability to grow on GB and sarcosine, thus suggesting that 2-79 has additional unidentified transporters involved in the uptake of these QACs.

**TABLE 1:** Ability of *P. synxantha* 2-79 and its isogenic mutants to utilize choline, glycine betaine, sarcosine, or carnitine as carbon sources^*a*^

Strain	Bacterial concentration (log CFU mL^−1^)							
	Choline		Glycine betaine		Sarcosine		Carnitine	
	Control	0.3M NaCl	Control	0.3M NaCl	Control	0.3M NaCl	Control	0.3M NaCl
Wild type	8.53 ± 0.02	8.30 ± 0.01	8.71 ± 0.01	8.60 ± 0.02	8.43 ± 0.02	8.51 ± 0.03	n.g.	n.g.
△betT1	8.38 ± 0.01	8.27 ± 0.01*	8.70 ± 0.01	8.60 ± 0.01	8.44 ± 0.02	8.42 ± 0.02	n.g.	n.g.
△betT2	8.45 ± 0.00	8.19 ± 0.00*	8.70 ± 0.00	8.60 ± 0.01	8.38 ± 0.01	8.39 ± 0.04	n.g.	n.g.
△betT3	8.47 ± 0.00	8.29 ± 0.00	8.70 ± 0.01	8.58 ± 0.01	8.42 ± 0.02	8.40 ± 0.02	n.g.	n.g.
△cbcXWV	n.g.	n.g.	8.67 ± 0.01*	8.70 ± 0.01	8.47 ± 0.03	8.44 ± 0.02	n.g.	n.g.
△opuC	8.43 ± 0.01	8.34 ± 0.01	8.70 ± 0.01	8.70 ± 0.02	8.43 ± 0.02	8.37 ± 0.03*	n.g.	n.g.
△betT1△betT2	8.27 ± 0.01*	8.31 ± 0.08	8.70 ± 0.01	8.75 ± 0.00	8.44 ± 0.02	8.39 ± 0.01	n.g.	n.g.
△betT1△betT2△cbcXWV	n.g.	n.g.	8.67 ± 0.01*	8.75 ± 0.01	8.47 ± 0.01	8.41 ± 0.03	n.g.	n.g.
△betT1△betT2△betT3 △cbcXWV	n.g.	n.g.	8.66 ± 0.02*	8.73 ± 0.01	8.50 ± 0.02	8.43 ± 0.04	n.g.	n.g.
△betT1△betT2△betT3 △cbcXWV △opuC	n.g.	n.g.	8.68 ± 0.01*	8.75 ± 0.02	8.45 ± 0.01	8.40 ± 0.02	n.g.	n.g.

^*a*^ The strains were cultured in half-strength 21C medium with 20 mM choline, glycine betaine, sarcosine, or carnitine used as a C source. The incubation was performed in control conditions or in the presence of 0.3 M NaCl. All cultures were incubated for 48 h at 27°C, and the maximum growth was measured by recording 600 nm, and the OD_600_ values were converted to log CFU ml^-1^. For individual treatments, asterisks indicate means that are significantly different from the wild type according to ANOVA followed by Tukey-Kramer’s HSD test (*P* < 0.05). n.g., no growth.

**Fig 6:**
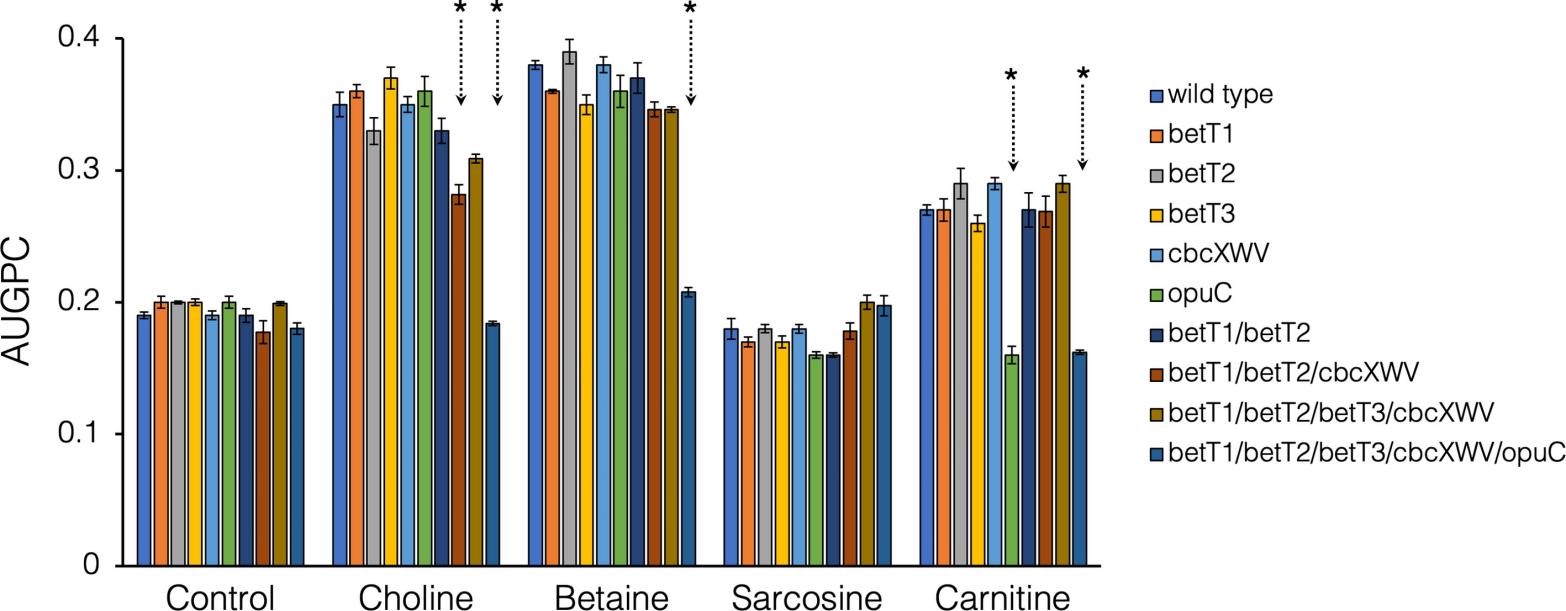
The effect of transporter gene mutations on the ability of *P. synxantha* 2-79 to use different QACs for osmoprotection. All strains were cultured in the half-strength 21C-glucose-pyruvate medium supplemented with a range of NaCl concentrations (i.e., 0.15, 0.3, 0.45, 0.6, 0.75, and 0.9 M). The growth medium was also supplemented with 1 mM choline, glycine betaine, sarcosine, or carnitine. All cultures were incubated for 48 h at 27°C, and the maximum growth was measured by recording 600 nm. The OD_600_ readings were plotted and converted to an area under the growth progress curve (AUGPC). Within each treatment, asterisks indicate AUGPC values that are significantly different according to ANOVA followed by Tukey-Kramer’s HSD test (*P* ≤ 0.05).

Under water stress and in the presence of an alternative C source (glucose or pyruvate), 2-79 used exogenous quaternary ammonium compounds for osmotic protection. Among the tested QACs, GB and choline functioned as the most effective osmoprotectants, whereas sarcosine was less effective, and its effect diminished at NaCl concentrations above 0.3 M (Fig. 6). The analysis of transporter mutants revealed that the uptake of carnitine for osmoprotection is provided exclusively by the OpuC transporter. The uptake of the other QACs likely requires a coordinated action of the BetT1, BetT2, BetT3, and OpuC systems, as evident from the decline in the ability to utilize choline and GB as osmoprotectants observed in multiple mutants. The reduced growth of the triple mutant (Δ*betT1*, Δ*betT2*, and Δ*cbcXWV*) suggests that *betT3* and *opuC* contribute to the uptake of choline. Finally, the multiple (Δ*betT1*/Δ*betT2*/Δ*betT3*/Δ*cbcXWV*/Δ*opuC*) mutant lost the ability to use any quaternary ammonium compound as an osmoprotectant.

We also employed ice nucleation bioreporter derivatives of 2-79 to probe the expression of *cbcX*, *opuC*, and *betT1* transporter genes during the microbial colonization of the host plant. The bioreporter strains were introduced in the rhizosphere of water-replete and water-stressed *B. distachyon* seedlings*,* and changes in the expression of target genes were monitored by measuring ice nucleation activity. Results of these assays revealed that all tested promoters were active in 2-79 colonizing plant roots (Fig. S5). The reporter assays also revealed that the *betT1* reporter was significantly upregulated in bacteria isolated from osmotically stressed plants, while *cbcX* was significantly induced under matric stress relative to controls carrying the empty vector.

### QAC uptake and biofilm pathways contribute to the rhizosphere fitness of 2-79

**Fig 7:**
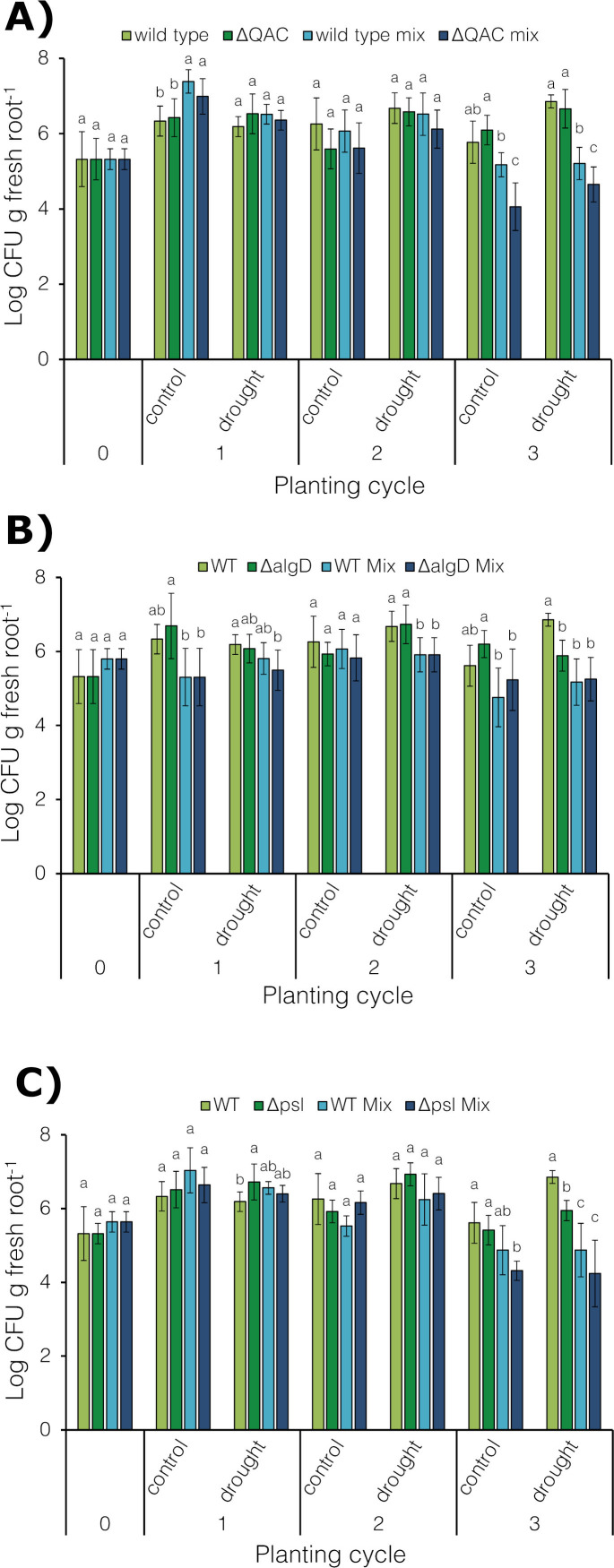
Rhizosphere fitness of wild-type 2-79 and its isogenic mutants lacking multiple QAC transporters (**A**), alginate (**B**), or Psl (**C**) exopolysaccharides. The plants were grown for three consecutive 3-week cycles, as described in the Materials and Methods. Each strain was introduced into the soil at a final density of approximately 10^5^ CFU per gram of soil (cycle 0) in single inoculations and approximately 0.5 × 10^5^ CFU per gram of soil in mixed inoculations (1:1 ratio). The bars represent means, and the error bars indicate standard deviations. Bars in the same cycle and water treatment followed by the same letter are not significantly different, as assessed by ANOVA followed by Tukey-Kramer’s HSD test (*P* > 0.05).

We further compared the relative fitness of the wild-type *P. synxantha* 2-79 and its multiple QAC transporter (Δ*betT1*/Δ*betT2*/Δ*betT3*/Δ*cbcWV*/Δ*opuC*) mutant in a competitive rhizosphere colonization cycling experiment with *B. distachyon* Bd21. The isogenic mutant was introduced into the potting mix either individually or as a mixed inoculation (1:1 ratio) with the wild-type strain. The inoculated potting mix was used to grow *B. distachyon* Bd21 for three successive 3-week cycles. Throughout the experiment, the water-replete controls were maintained at 80% FC, whereas drought-stressed plants were kept at ~40% FC, and the rhizosphere populations of bacteria were enumerated after each cycle. The results of the plant assays revealed that rhizosphere population densities of the QAC transporter mutant were similar to wild-type 2-79 after the first cycle, and they increased by about one log, especially under well-watered conditions (Fig. 7A). In the second cycle, the levels of the mutant in the plant rhizosphere densities started to decline both in the single and mixed inoculation treatments. In the third cycle, the single inoculation densities of the parental strain and the mutant remained similar at both water treatment regimens. In contrast, in the mixed inoculations, the rhizosphere population levels of the QAC transporter mutant dropped significantly both in the control and drought-stressed conditions, indicating a decline in the rhizosphere fitness relative to the wild-type 2-79.

Similar to the QAC transporter mutant, the colonization densities of the alginate (Δ*algD*) mutant fluctuated throughout the cycling experiment (Fig. 7B). In individual inoculations, the population densities of the Δ*algD* mutant were closely comparable to the wild-type strain during the first two cycles under both soil moisture conditions. A similar trend was observed in the third cycle for the well-watered control, but under drought stress conditions, the wild-type strain 2-79 outcompeted its isogenic alginate mutant. In all mixed inoculation treatments, the population densities of both strains remained similar across all sampling points.

For the Psl mutant, the population densities matched those of the wild-type strain during the first two cycles, in both single and mixed inoculations and under both soil moisture levels (Fig. 7C). However, in the third cycle, the Δ*psl* mutant colonized *Brachypodium* roots significantly less efficiently than the wild-type 2-79 in individual inoculations on drought-stressed plants. This trend was also observed, though not significantly, in the mixed inoculations, regardless of the soil moisture level.

## DISCUSSION

Our previous studies revealed an abundance of 2-79-like bacteria in the rhizosphere of wheat grown in parts of central and eastern Washington, which receive annual precipitation as low as 150–300 mm (17, 54, 55). These bacteria produce the broad-spectrum antibiotic phenazine-1-carboxylic acid (PCA) and control *Rhizoctonia solani* AG-8, a ubiquitous soilborne fungal pathogen representing major yield constraints in wheat grown in this semi-arid region (56). Interestingly, the population density of the 2-79-like pseudomonads was inversely related to annual precipitation or irrigation, suggesting that soil moisture represents a significant abiotic factor driving the development of these rhizobacteria and that they are adapted to the rhizosphere of cereals growing in areas of low precipitation (18, 57).

We hypothesized that the enrichment of *P. synxantha* 2-79-like bacteria in the Pacific Northwest soils is mediated, in part, by changes in root exudation in dryland cereals. Plants attract and support complex microbial communities by releasing a substantial amount of photosynthetically fixed carbon through their roots in the form of exudates that contain diverse simple and complex sugars, sugar alcohols, amino acids, organic, aliphatic, and fatty acids, sterols, and phenolics (58–61). Abiotic stressors, such as drought, can alter the quantity and composition of root exudates (62). These stress-driven changes in exudate profiles enable plants to recruit and influence beneficial microorganisms that help mitigate stress by promoting nutrient uptake, producing protective compounds, or enhancing stress tolerance, a phenomenon known as the “cry-for-help hypothesis” (63). In agreement with this hypothesis, our studies revealed that subjecting wheat to water stress significantly alters the levels of multiple primary and secondary metabolites in root exudates. Significantly, the osmotically challenged plants secreted elevated amounts of the QACs choline and glycine betaine (GB), which are highly soluble and neutrally charged metabolites that, when accumulated at sufficient levels, can help to maintain turgor and stabilize cellular components, thus protecting microorganisms from water stress (64, 65). The exudates markedly improved the growth of water-stressed strain 2-79 and altered the expression of its QAC metabolism genes.

The ability to import choline and to either catabolize or convert it to the osmoprotectant GB is widespread among soil bacteria and represents a preferred way of counteracting water stress compared to *de novo* synthesis, since it helps to save carbon and energy resources. Pseudomonads take up QACs via transporters of the betaine-choline-carnitine transporter (BCCT) family, which are powered by proton/sodium-motive force and ATP-binding cassette (ABC) transporters energized by ATP (66). The plant pathogen *P. syringae* uses the ABC transporter OpuC to take up choline, GB, choline-O-sulfate, and carnitine under high osmotic stress, while the BCCT transporter BetT is used for the uptake of choline and acetylcholine under both high and low osmotic conditions (14, 67). *Pseudomonas syringae* also has a second ABC transporter, CbcWV, which operates to take up choline, GB, and carnitine as a nutrient source under low osmolarity conditions. Although OpuC and CbcWV are also present in *P. aeruginosa* and *P. putida*, these species encode three BCCT transporters, termed BetT1, BetT2, and BetT3. Studies in *P. aeruginosa* revealed that BetT1 or BetT3 prime the induction of CbcXWV, which subsequently functions, in combination with BetT1, as a primary transporter for the uptake of choline under low osmolarity (51). Under hyperosmolar conditions, the ABC transporter OpuC and BetT3 act to import QACs for osmoprotection (66). Genes involved in the transport and catabolism of QACs are regulated by choline-responsive repressor BetI and GbdR, an AraC-family activator that senses GB and dimethylglycine (53). Despite the overall conservation of QAC metabolism in pseudomonads, we observed several genes that were variably distributed in members of the *P. fluorescens* group. For example, cross-genome comparisons revealed that the number of BCCT family transporters in species of the *P. fluorescens* complex varied between one and three (Fig. S6). In contrast, strains of *P. syringae* had only one BCCT transporter, while *P. putida* carried up to six such genes. Another variably present gene encoded gamma-butyrobetaine hydroxylase, an enzyme that catalyzes the formation of L-carnitine from gamma-butyrobetaine (Fig. S2). These variations in the number and location of QA transport and catabolic genes are consistent with the presence of strain- and species-specific differences in the arrangement of regulatory networks that govern the uptake and catabolism of quaternary amines. We speculate that such genetic differences represent niche-specific adaptations that allow individual strains to exploit microniches in the rhizosphere and/or better adapt to different soils and plant hosts.

Overall, *P. synxantha* 2-79 closely resembles *P. aeruginosa* in the arrangement of genes involved in the uptake and catabolism of QACs and likely in the regulation of these pathways (Fig. S7). Under hyperosmolar conditions, 2-79 uses choline, GB, and carnitine as osmoprotectants. The analysis of transporter mutants suggests that under water-stressed conditions, the BetT transporters, together with OpuC, function to take up choline and GB, while the import of carnitine occurs via OpuC. In contrast, under low osmolarity, 2-79 utilizes choline, GB, and sarcosine as carbon sources, and the uptake of choline occurs via CbcWV. The loss of all QAC transporters did not affect the growth on GB or sarcosine, suggesting the involvement of other transporter(s) and illustrating an impressive redundancy in QAC uptake pathways of 2-79. It is plausible that GB and sarcosine can also be taken up by the MFS transporters C4K02_0724 and C4K02_0473, which are encoded by genes associated with putative GbdR-binding sites.

QACs are common in plants, particularly choline, which is synthesized in the cytosol and incorporated into the polar head group of phosphatidylcholine, an essential phospholipid component of plant membranes (68, 69). Choline also acts as a precursor for GB, an important compatible solute that supports plant adaptation to saline and drought conditions (70). Its accumulation helps maintain osmotic balance, mitigates oxidative damage caused by reactive oxygen species, and stabilizes photosystem II (71). Moreover, GB boosts overall plant growth and reproductive success, both in normal and stressed conditions, by regulating stress hormones and enhancing ion homeostasis. The distribution of GB in plants varies by tissue and developmental stage, with higher accumulation observed in younger leaves, especially under water stress (72, 73). This pattern reflects GB synthesis in older leaves and its translocation to younger tissues through a source-to-sink mechanism regulated by hormonal signals to support growth and improve stress tolerance. Although root GB levels are generally lower than in leaves, localized buildup of this metabolite in the pericycle, a key tissue for water and ion regulation, suggests a protective role during osmotic stress (74). Along with tissue-level accumulation, quaternary ammonium compounds (QACs), such as GB and choline, are also secreted into the rhizosphere and have been detected in root exudates of various plant species, including *Brachypodium distachyon*, *Arabidopsis thaliana*, *Avena barbata*, and legumes (19, 75–77).

**Fig 8:**
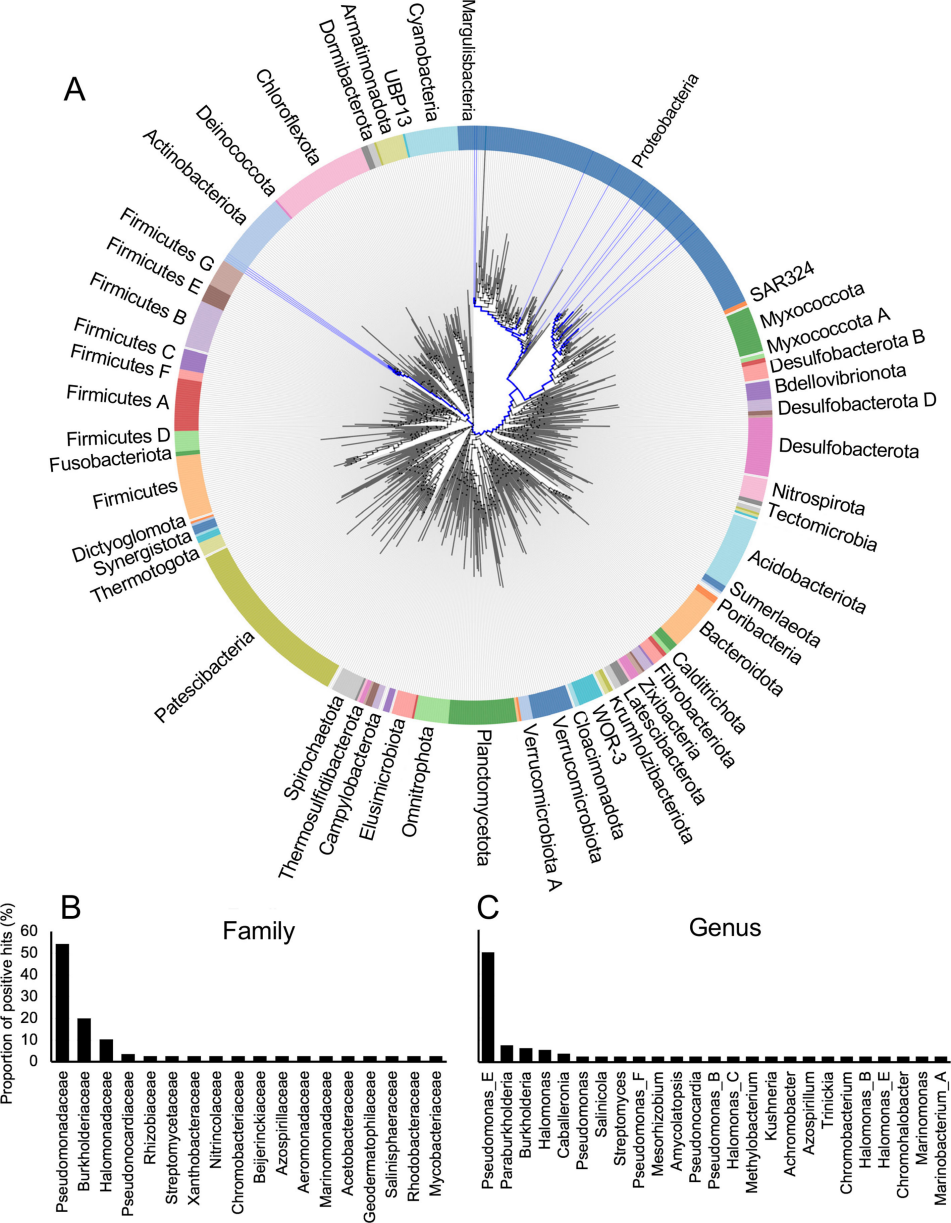
The distribution of QAC catabolism pathways across different bacterial phyla (**A**) and the proportion of bacterial families (**B**) and genera (**C**) that possess *betAB*, *gbcAB*, *dgcAB,* and *soxBDAG* genes. The analysis was performed in AnnoTree (32) using the following cutoff parameters: % identity >30, *e*-value <0.00001, % subject alignment >70, and % query alignment >70.

The utilization of plant-derived choline and GB plays an important role in the colonization of leaf tissues by the plant pathogen *P. syringae* (15) and the human enteric pathogen *Escherichia coli* O157:H7 (78). Our results expand these findings by revealing that the utilization of plant-derived QACs plays an important role in the ability of *P. synxantha* 2-79 to persist in the water-stressed rhizosphere. More broadly, the widespread presence of QAC catabolism genes in diverse Gram-negative and Gram-positive bacteria, including other species of *Pseudomonas*, *Burkholderia*, *Halomonas,* and *Streptomyces* (Fig. 8), suggests that the ability to take up and metabolize these compounds represents an important adaptation to the rhizosphere lifestyle.

In addition to the ability to take up and metabolize plant-derived osmoprotectants, 2-79 has evolved an impressive array of genes predicted to function in the *de novo* synthesis of the compatible solutes *N*-acetyl-glutaminyl glutamine amide (NAGGN), L-ectoine, and trehalose (Table S3). The importance of NAGGN and trehalose biosynthesis genes for the osmotic stress response was evident from the severe impairment in the growth of the corresponding mutant strains compared to that of the wild-type strain when the osmolarity of the medium was elevated with NaCl. In addition to synthesizing compatible solutes, many bacteria cope with the deleterious effects of water stress by forming biofilms that function as hydrating agents under water-limited conditions (79–81). *Pseudomonas* biofilms are embedded in a matrix composed of extracellular DNA, proteins, lipids, and various extracellular polysaccharides, some of which serve structural roles, while others play a minor part in biofilm formation but are crucial for stress protection (82, 83). Like many other members of the *P. fluorescens* group (84), 2-79 carries gene clusters involved in the production of alginate, a β−1,4 linked copolymer of *O*-acetylated D-mannuronic and L-glucuronic acid, and Psl, a polysaccharide composed of D-mannose, D-glucose, and L-rhamnose. The strain does not produce cellulose or other known exopolysaccharides, such as Pel, Peb, or Pea. In plant-associated pseudomonads, alginate plays a minor structural role in biofilm formation but contributes to epiphytic and rhizosphere fitness (85, 86). Alginate also contributes to the survival of *P. fluoresc*ens in moist and dry soils (87) and protects *P. putida* from water stress by contributing to biofilm architecture and attenuating the accumulation of reactive oxygen species generated under desiccation (88, 89).

In contrast to alginate, Psl is a structural polysaccharide that plays a crucial role in biofilm formation by facilitating initial adherence to surfaces, promoting cell-to-cell aggregation, and contributing to the structural stability of the biofilm matrix (10). In *P. syringae*, the Psl-like polysaccharide is involved in adhesion to plant surfaces and supports the bacterium’s ability to resist desiccation, thereby promoting its survival in harsh environmental conditions (90). This polysaccharide is also involved in the colonization of plant roots by *P. chlororaphis*, and mutants lacking Psl exhibited impaired attachment and reduced biocontrol efficacy (91). Our results agree with these findings by demonstrating that the alginate- and Psl-deficient mutants colonized the plant rhizosphere less efficiently than the parental strain. The effect was especially pronounced in the Psl mutant, indicating the importance of this polysaccharide for the interaction of 2-79 with the host plant under both control and in water-stressed conditions.

Our findings also reveal that 2-79 can finely tune its metabolic and other cellular processes in response to changes in the composition of root exudates. Many pathways affected by root exudates function in the uptake and breakdown of diverse nutrients such as carbohydrates, amino acids, and phenolics and are crucial for energy production and growth, while other pathways are involved in protein turnover, metal ion homeostasis, efflux processes, and flagellum function. Collectively, these transcriptome responses signify an adjustment of metabolism to utilize available resources while adapting to stress conditions.

## Data Availability

Sequencing data from this study have been deposited in the NCBI database under BioProject PRJNA1244843.

## SUPPLEMENTAL MATERIAL

Supplemental figures aem.00768-25-s0001.pdf Figures S1 to S7.

Supplemental references aem.00768-25-s0002.pdf References for supplemental materials.

Table S1 aem.00768-25-s0003.docx Oligonucleotide primers used in this study.

Table S2 aem.00768-25-s0004.xlsx RNA-seq data.

Table S3 aem.00768-25-s0005.docx Predicted functions of putative water stress response genes in *P. synxantha* 2-79 and their homologs in selected rhizosphere strains of the *P. fluorescens* group.

ASM does not own the copyrights to Supplemental Material that may be linked to, or accessed through, an article. The authors have granted ASM a non-exclusive, world-wide license to publish the Supplemental Material files. Please contact the corresponding author directly for reuse.
