# Supplementary material for: Root exudates protect rhizosphere Pseudomonas from water stress
Source: Appl Environ Microbiol. 2025 Aug 5;91(9):e00768-25. doi: 10.1128/aem.00768-25 (PMC12442406; doi:10.1128/aem.00768-25)
Supplement: Supplemental figures — Figures S1 to S7. [file aem.00768-25-s0001.pdf]

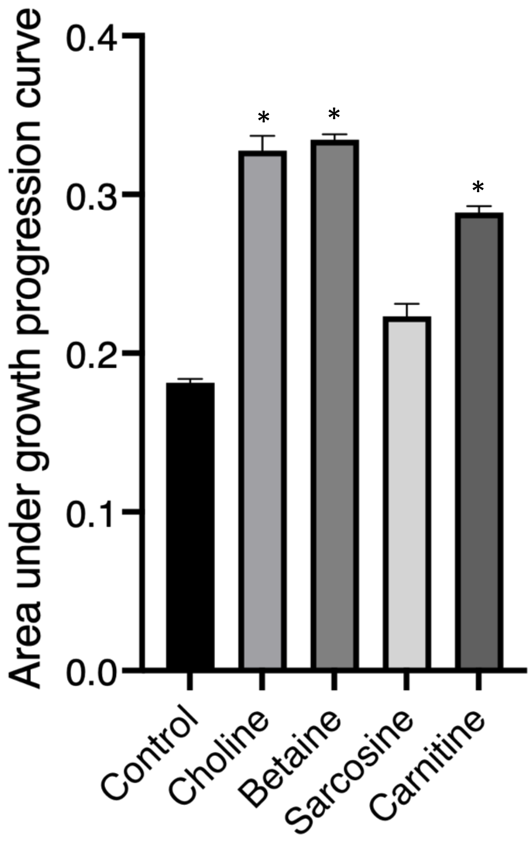

Supplementary Figure 1. The protective effect of different QACs on the growth of *P. synxantha* 2-79 under osmotic stress. The bacteria were cultured in ½-21C-glucose-pyruvate medium with 1 mM QACs and varying NaCl levels (0, 0.15, 0.3, 0.45, 0.6, 0.75, and 0.9 M). All cultures were incubated for 48 hours at 27°C, and the maximum growth was measured by recording at 600 nm. The OD_{600} values were plotted and converted to an area under the growth progress curve (AUGPC). Asterisks indicate significant differences from the QAC-free control, as analyzed by the Kruskal-Wallis test with Dunn's post hoc test for multiple comparisons ($P < 0.05$).

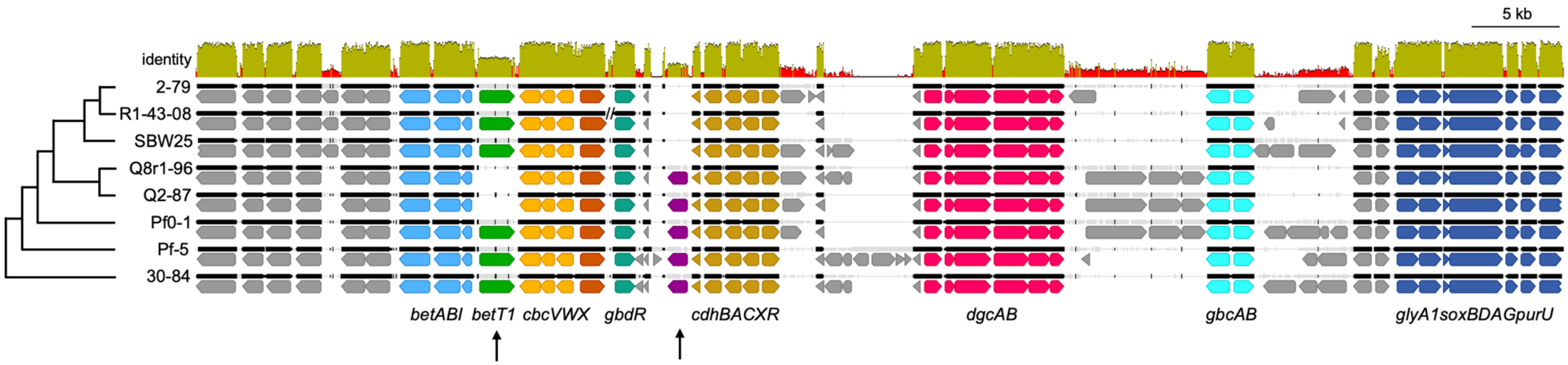

Supplementary Figure 2. Synteny of genome regions carrying genes involved in the uptake and catabolism of quaternary amines in 2-79 and selected strains of the *P. fluorescens* group. DNA sequences were aligned using MAFFT (Katoh & Standley, 2013). Predicted genes and their orientations are indicated by horizontal arrows, with homologous QAC metabolism genes highlighted in color, and other genes shown in grey. Thin vertical arrows at the bottom indicate the variable presence of genes for the BCCT transporter BetT1 and gamma-butyrobetaine dioxygenase. All genes and intergenic regions are to scale, as shown by the scale bar in the upper right corner.

A)

Gene	Function	Motif Position	Strand	Motif Sequence
betX	Substrate-binding protein	(2022888-2022918)	+	GTCGTTTCTTGCTCAGCCGAGGTCGCTGGCA-
C4K02_5598	Hypothetical protein	(6103627-6103657)	-	GGCGTGATCAAGACGCAAGCGGTCGCGCCCG-
cbcX	Substrate-binding protein	(5968570-5968600)	-	GTCGTATCCAGTACAGGGAGGGTCGCATCCA-
cdhX	Substrate-binding protein	(5961115-5961145)	+	GTCGCTGCAGTGCAAGAGTCGGTCGCCGCCG-
C4K02_5456	Membrane dipeptidase	(5957236-5957266)	-	GACGCTTTCGGCAATTCCCCTGTCGTTTTTG-
gbcB	Rieske 2Fe-2S family protein	(5945498-5945528)	-	GTCGCCGTAAGTATGTGA-ATGTCGCAGATAG
gbcA	Ferredoxin-NADP reductase	(5945555-5945585)	+	GTCGGGATCAGATTGGGCCATGTCGCCCATG-
C4K02_0724	MFS transporter	(812116-812146)	+	GTCGTTCAGGGTTTCAGTCAGGTCGCATCCA-
C4K02_5417	Gly cleavage system protein R	(5909595-5909625)	-	GTCGTTTAGCGACCCACTTTGGTCGTACGGT-
C4K02_0473	MFS transporter	(520232-520262)	-	GTCGTAAAACCTATCAAACAGGTCGAACAAT-
C4K02_4729	Hypothetical protein	(5122846-5122876)	+	GTCGCTACTGGTTACTAT-GGGTCGGTTTCAG
C4K02_1929	acyl-CoA dehydrogenase	(2096885-209691)	-	GTCGGCGCCTGTGGATGGGTAGTCGAGTACA-
gabP	GABA permease	(404176-404206)	+	GTCGCATCGGCACAGTAGCGTGTCGCGCCAT-
argG	Argininosuccinate synthase	(1385006-1385036)	+	GTCGCTTTATCTTCATTTGCTGTCGCGCCCC-
C4K02_5067	Hypothetical protein	(5501950 - 5501980)	-	GTCGCCTCAGTGCGCAAAACAGTCGTCTGGG-

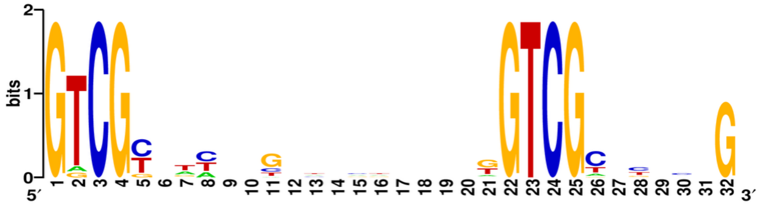

B)

	Gene	Function	Genome Position	Strand	Motif Sequence
P.synxantha 2-79	*betT1*	BCCT transporter	(5974202 - 5974220)	-	GATTGAACGTTCAATCAAC
P.aeruginosa	BetI binding site				TATTGAACGTTCAATCAAC
E.coli	BetI binding site				TATTGATTGGACGTTCAAT
E.meliloti	BetI binding site				TATTGATTGATGGATCAAT

Supplementary Figure 3. Putative binding sites for GbdR and BetI transcriptional regulators in the *P. synxantha* 2-79 genome. A) GbdR gene targets, their functions, genome locations, and multiple sequence alignment of putative binding sites, with conserved residues highlighted in red. The binding site sequences were aligned using ClustalW, and the consensus GbdR binding sequence was generated using WebLogo 3 (Crooks et al., 2004). B) The putative BetI binding site in the promoter region of *betT1* in *P. synxantha* 2-79 and its alignment with known BetI binding sites from *P. aeruginosa*, *Escherichia coli*, and *Ensifer meliloti*.

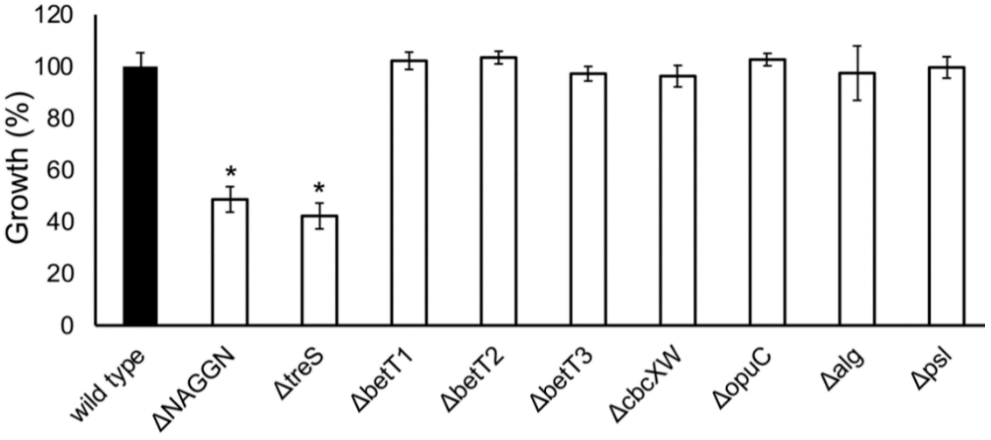

Supplementary Figure 4. The contribution of pathways involved in the synthesis of NAGGN and trehalose, uptake of QACs (*betT1, betT2, betT3, cbcXW, opuC*), and production of biofilm matrices (*alg, psl*) to the ability of 2-79 to resist osmotic stress. The strains were grown in low osmoticum ½-21C-glucose-pyruvate medium supplemented with 0.3 M NaCl, and bacterial growth was measured by recording OD_{600} after 48 hours of incubation at 27°C. The growth achieved by the wild type 2-79 was set at 100%. Asterisks indicate significant differences as analyzed by the Kruskal-Wallis test with Dunn's post hoc test for multiple comparisons ($P < 0.05$).

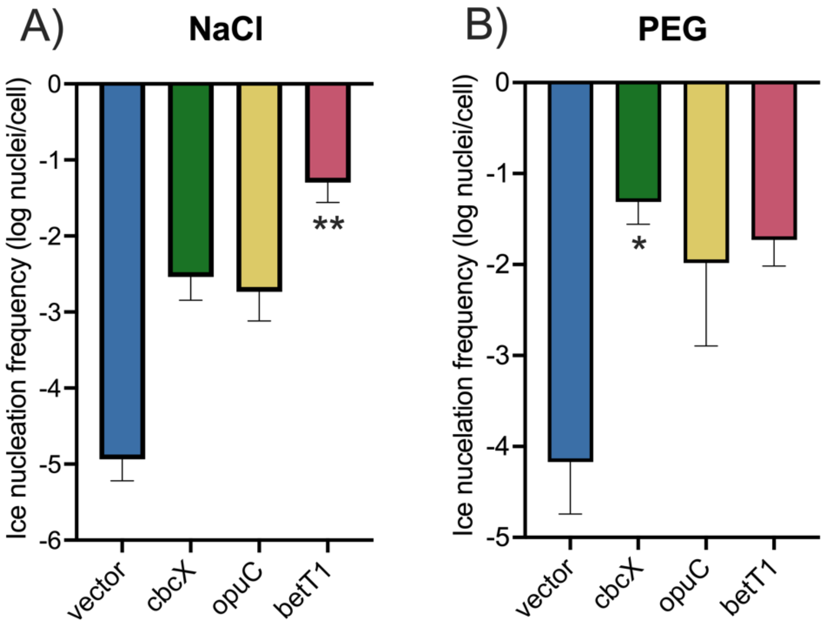

Supplementary Figure 5. Ice nucleation activity of *cbcX*-, *opuC*-, and *betT1-inaZ* reporter fusions on roots of *B. distachyon* Bd21 challenged with NaCl or PEG 8000. Ice nucleation frequency was determined on a per-cell basis, as described in Materials and Methods. Asterisks indicate significant differences from the QAC-free control as analyzed by the Kruskal-Wallis test with Dunn's post hoc test for multiple comparisons (*P* < 0.05).

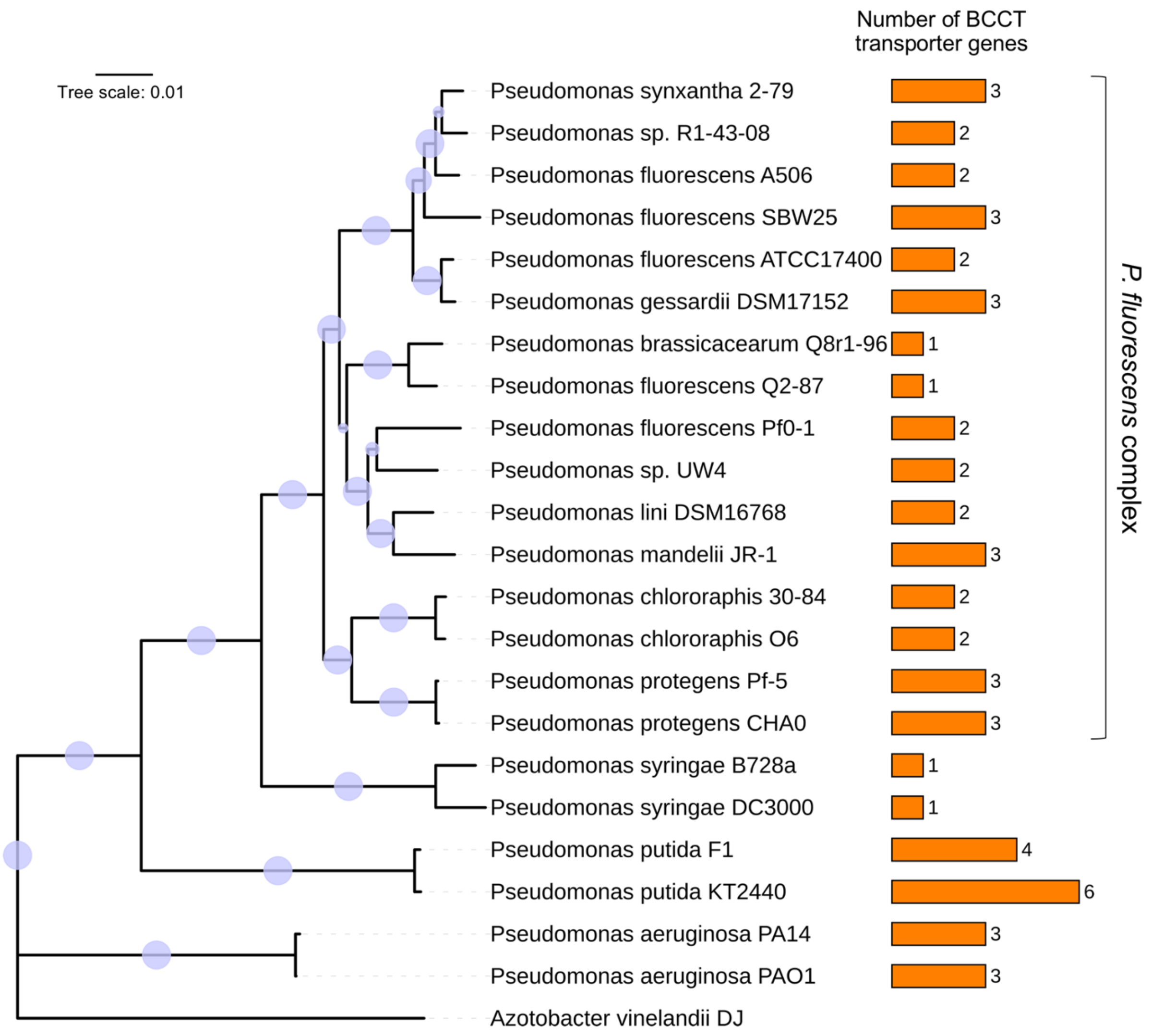

Supplementary Figure 6. The variable number of BCCT transporter genes in species of the *P. fluorescens* complex, as well as strains of *P. syringae*, *P. putida,* and *P. aeruginosa*. The MLSA phylogeny was established based on concatenated protein sequences of AcsA (acetyl-CoA synthetase), DnaE (α subunit of DNA polymerase III), GuaA (GNP synthase), GyrB (subunit B of DNA gyrase), MutL (DNA mismatch repair protein), PpsA (phosphoenolpyruvate synthase), PyrC (dihydroorotase), RecA (recombinase RecA), and RpoB (β subunit of RNA polymerase). Homologs from *Azotobacter vinelandii* DJ were utilized as an outgroup. Sequences were aligned using MAFFT 7.309 (Katoh & Standley, 2013), and the phylogenetic tree was constructed using the neighbor-joining (NJ) algorithm. Blue circles on the tree nodes represent bootstrap values, ranging from 80% (the smallest circle) to 100% (the largest circle).

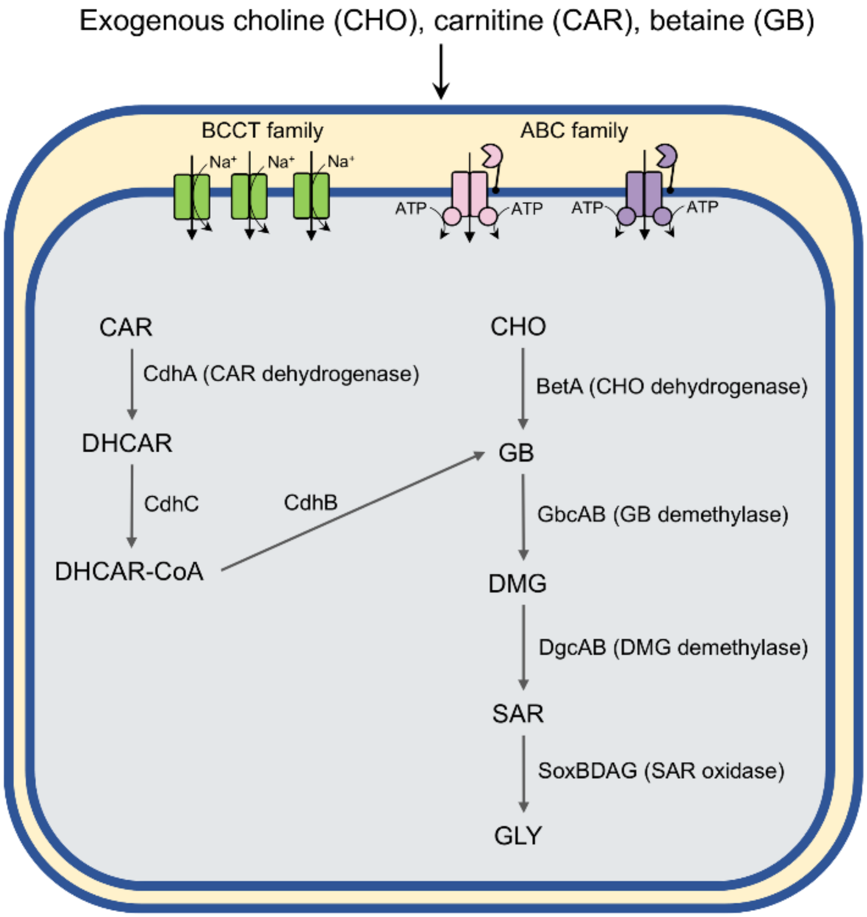

Supplementary Figure 7. A summary of *P. synxantha* 2-79 genes predicted to be involved in the uptake and catabolism of QACs. DHCAR; 3-dehydro carnitine, DHCAR-CoA; 3-dehydrocarnitine-CoA; DMG, dimethylglycine; SAR, sarcosine; GLY, glycine.
