# Supplementary material for: Root exudates protect rhizosphere Pseudomonas from water stress
Source: Appl Environ Microbiol. 2025 Aug 5;91(9):e00768-25. doi: 10.1128/aem.00768-25 (PMC12442406; doi:10.1128/aem.00768-25)
Supplement: Supplemental references — References for supplemental materials. [file aem.00768-25-s0002.pdf]

References for supplemental material:

1) Crooks GE, Hon G, Chandonia JM, Brenner SE. 2004. WebLogo: A sequence logo generator, Genome Res 14:1188-1190.

2) Green MR, Sambrook J. 2012. Molecular Cloning: A Laboratory Manual, 4th ed. Cold Spring Harbor Laboratory Press, Cold Spring Harbor, N.Y.

3) Katoh K, Standley DM. 2013. MAFFT multiple sequence alignment software version 7: improvements in performance and usability. Mol Biol Evol 30(4):772-780.

4) Mavrodi DV, Bonsall RF, Delaney SM, Soule MJ, Phillips G, Thomashow LS. 2001. Functional analysis of genes for biosynthesis of pyocyanin and phenazine-1-carboxamide from *Pseudomonas aeruginosa* PAO1. J Bacteriol 183:6454-6465.
