# Supplementary material for: Root exudates protect rhizosphere Pseudomonas from water stress
Source: Appl Environ Microbiol. 2025 Aug 5;91(9):e00768-25. doi: 10.1128/aem.00768-25 (PMC12442406; doi:10.1128/aem.00768-25)
Supplement: Table S1 — Oligonucleotide primers used in this study. [file aem.00768-25-s0003.docx]

**Supplementary Table 1.** Oligonucleotide primers used in this study.

| Primer | Sequence | Reference |
| --- | --- | --- |
| M13f (-47) | 5’-CGCCAGGGTTTTCCCAGTCACGAC-3’ | Green & Sambrook, 2012 |
| M13r (-48) | 5’-AGCGGATAACAATTTCACACAGGA-3’ | Green & Sambrook, 2012 |
| betT1-1 | 5'-CAGGGTTTTCCCAGTCACGACCAAGTGCATAAGT GGTTGGCG-3' | This study |
| betT1-2 | 5'-CGACGCAGATGTTACTCACTTAAACAACAGAATC AGCGCGGTG-3' | This study |
| betT1-3 | 5'-TAAGTGAGTAACATCTGCGTCGGAAGAACGCAC CCGCTACTAC-3’ | This study |
| betT1F | 5'-CAGGAATTGGGGATCGGATGCATACCGACCTTGG GCATG-3' | This study |
| betT1RinaZ | 5'-ATAGATCCTTTGGGGTTAAGCCGTATAAGAGAGG CAGAACTCATGGC-3 | This study |
| betT2-1 | 5'-CAGGGTTTTCCCAGTCACGACGGCCCGAGCTTCT TTATCGACC-3' | This study |
| betT2-2 | 5'-CGACGCAGATGTTACTCACTTACCAAACAGCAGG ATAAAAGTCG-3' | This study |
| betT2-3 | 5'-TAAGTGAGTAACATCTGCGTCGGAGCATCCGTTT ATCTACCAGG-3' | This study |
| betT2-4 | 5'-GGATAACAATTTCACACAGGACCAAAGTTGATCT CGGCGCTGC-3' | This study |
| betT3-1 | 5'-CAGGGTTTTCCCAGTCACGACTGCTGGTGTTGGT GGTGGACGA-3' | This study |
| betT3-2 | 5'-CGACGCAGATGTTACTCACTTACATACAGGCCTC CTTGGAAATG-3' | This study |
| betT3-3 | 5'-TAAGTGAGTAACATCTGCGTCGATTGCCATTGCCCTGTTGCTTG-3' | This study |
| betT3-4 | 5'-GGATAACAATTTCACACAGGAAGCCTGCAAAAGT TCTATCGCG-3' | This study |
| opuC1 | 5'-CAGGGTTTTCCCAGTCACGACTCAACACGATCGA ACTGTACCG-3' | This study |
| opuC2 | 5'-TAAGTGAGTAACATCTGCGTCGTTCCTTTGCCAA GCCTTAAGAC-3' | This study |
| opuC3 | 5'-CGACGCAGATGTTACTCACTTAGTTCTTCGAGAT GCAGCGCGCG-3' | This study |
| opuC4 | 5'-GGATAACAATTTCACACAGGAGAACACCCGCTCT GCATCCATC-3' | This study |
| opuCF | 5'-CAGGAATTGGGGATCGGAGGTCGGATTTGCGCGA TTTCAC-3' | This study |
| opuCRinaZ | 5'-ATAGATCCTTTGGGGTTAAGCCGACTAGGACGAG AATCAGATGAGC-3' | This study |
| cbcXWV1 | 5'-CAGGGTTTTCCCAGTCACGACATGCGACCCTCCC TGTACTG-3' | This study |
| cbcXWV2 | 5'-CGACGCAGATGTTACTCACTTACCAGCTCGTCCT TGAACTTGGC-3' | This study |
| cbcXWV3 | 5'-TAAGTGAGTAACATCTGCGTCGGCGACTCATGGC TGGACCTG-3' | This study |
| cbcXWV4 | 5'-GGATAACAATTTCACACAGGACCCGGGATGTGCT CGATCAG-3' | This study |
| cbcXF | 5'-CAGGAATTGGGGATCGGACTTGAACGAGGTGCT ACGCTCAG-3' | This study |
| cbcXRinaZ | 5'-ATAGATCCTTTGGGGTTAAGCCGGGAGAAATTGA CGGTCTTGCAC-3' | This study |
| NAGGN1 | 5'-CAGGGTTTTCCCAGTCACGACCGGCAAATTGGAC GGCACTC-3' | This study |
| NAGGN2 | 5'-CGACGCAGATGTTACTCACTTAACTCGCCAGCTA ATCCACAC-3' | This study |
| NAGGN3 | 5’-TAAGTGAGTAACATCTGCGTCGACAGCCAACGCT TACTCAAG-3’ | This study |
| NAGGN4 | 5'-GGATAACAATTTCACACAGGACACAGGGTCATGC TGATGGC-3' | This study |
| treS1 | 5'-CAGGGTTTTCCCAGTCACGACGGTAGTGAAGAGG GCGGTC-3' | This study |
| treS2 | 5’-CGACGCAGATGTTACTCACTTAGGTGTTGCGCCA GGTGAAG-3’ | This study |
| treS3 | 5'-TAAGTGAGTAACATCTGCGTCGCTGGGGGAAAT GGACCTCT-3' | This study |
| treS4 | 5'-GGATAACAATTTCACACAGGACCCACGTGCAGCT CATAGAG-3' | This study |
| alg1 | 5'-CAGGGTTTTCCCAGTCACGACCTGTGCCGGTTGC CTGTCTG-3' | This study |
| alg2 | 5'-TAAGTGAGTAACATCTGCGTCGTCATCGGTGGCA GGTCGTAG-3' | This study |
| alg3 | 5'-CGACGCAGATGTTACTCACTTAGTTGTTCTTCCTC AGCCTGC-3' | This study |
| alg4 | 5'-GGATAACAATTTCACACAGGAGCCGAACAACTG GAACCAAG-3' | This study |
| psl1 | 5'-CAGGGTTTTCCCAGTCACGACTGCAACTGGACCC GCTCAATG-3' | This study |
| psl2 | 5'-TAAGTGAGTAACATCTGCGTCGGGCACTCAAGAG GCTGAACAG-3' | This study |
| psl3 | 5'-CGACGCAGATGTTACTCACTTAAACACGACCACC AACATCGAC-3' | This study |
| psl4 | 5'-GGATAACAATTTCACACAGGAGCACGCCATTGAT CACTTCGC-3' | This study |
| Stop | 5’-CGACGCAGATGTTACTCACTTA-3’ | This study |
| SAC1 | 5'-GATGTTTTCTTGCCTTTGATGTTC-3' | Mavrodi et al., 2001 |
| SAC2 | 5'-GTCTTTGCATTAGCCGGAGATC-3' | Mavrodi et al., 2001 |
| GM-UP | 5'-GGTGGCTCAAGTATGGGCATCA-3' | Mavrodi et al., 2001 |
| GM-LOW | 5'-ATAGAGAGCCACTGCGGGATCG-3' | Mavrodi et al., 2001 |
| soxBf | 5’-ATCGAATCCCACCCGCTGCAA-3’ | This study |
| soxBr | 5’-CCGATCACCAGGTCGCCCTT-3’ | This study |
| gbcAf | 5’-CTGCCGCACTCCTGGAACCAC-3’ | This study |
| gbcAr | 5’-GCGTCCTTGTGCACGATCCACT-3’ | This study |
| dgcAf | 5’-GCCAACGTCATCCCGAACATGAGC-3’ | This study |
| dgcAr | 5’-CAGCACCGGAACCTTGACCACT-3’ | This study |
| rpoDf | 5’-TCGTGGCAACAAGCAGGCAATCG-3’ | This study |
| rpoDr | 5’-CGCGCTCAACCAGGCCTTCGAAC-3’ | This study |
