# Supplementary material for: Root exudates protect rhizosphere Pseudomonas from water stress
Source: Appl Environ Microbiol. 2025 Aug 5;91(9):e00768-25. doi: 10.1128/aem.00768-25 (PMC12442406; doi:10.1128/aem.00768-25)
Supplement: Table S3 — Predicted functions of putative water stress response genes in P. synxantha 2-79 and their homologs in selected rhizosphere strains of the P. fluorescens group. [file aem.00768-25-s0005.docx]

**Supplementary Table 3.** Predicted functions of putative water stress response genes in *P. synxantha* 2-79 and their homologs in selected rhizosphere strains of the *P. fluorescens* group.

| 2-79 gene ID*^a^* | Gene name | | Predicted product function | Homolog in the genome of (blast e-value (% identity)): *^b^* | | | | | | |
| --- | --- | --- | --- | --- | --- | --- | --- | --- | --- | --- |
|  |  |  |  | R1-43-08 | SBW25 | Pf0-1 | Q8r1-96 | Q2-87 | Pf-5 | 30-84 |
| Pathway: trehalose biosynthesis | | | | | | | | | | |
| C4K02_2616 | *glgE* | α-1,4-glucan:maltose-1-phosphate maltosyltransferase (EC 3.2.1.1) | | 0.0 (95.2) | 0.0 (95.0) | 0.0 (81.3) | 0.0 (83.9) | 0.0 (83.9) | 0.0 (83.7) | 0.0 (84.6) |
| C4K02_2617 | *treS* | maltose alpha-D-glucosyl transferase/α-amylase (EC 5.4.99.16) | | 0.0 (95.6) | 0.0 (94.2) | 0.0 (85.8) | 0.0 (85.4) | 0.0 (85.2) | 0.0 (83.4) | 0.0 (84.8) |
| C4K02_2618 | *glgB* | 1,4-α-glucan branching enzyme (EC 2.4.1.18) | | 0.0 (97.2) | 0.0 (93.7) | 0.0 (84.8) | 0.0 (84.7) | 0.0 (84.3) | 0.0 (84.8) | 0.0 (85.6) |
| C4K02_3040 |  | Nucleotide-diphospho-sugar transferase | | 2e^-24^ (71.4) | 4e^-132^ (84.7) | 9e^-111^ (72.8) |  |  | 2e^-108^ (72.1) |  |
| C4K02_3039 |  | S-adenosyl-L-methionine-dependent methyltransferase | | 1.6e^-3^ (83.3) | 0.0 (77.9) | 0.0 (64.8) |  |  | 0.0 (63.8) |  |
| C4K02_3038 |  | LmbE family N-acetyl glucosaminyl deacetylase | | 0.0 (91.7) | 0.0 (89.7) | 0.0 (72.6) |  |  | 0.0 (69.4) | 0.0 (62.4) |
| C4K02_3037 |  | hypothetical protein | | 0.0 (86.9) | 0.0 (87.0) | 0.0 (66.2) |  |  | 0.0 (62.6) |  |
| C4K02_3036 | *glgX* | Glycogen debranching enzyme | | 0.0 (97.8) | 0.0 (96.5) | 0.0 (89.7) | 0.0 (90.1) | 0.0 (90.5) | 0.0 (91.2) | 0.0 (88.8) |
| C4K02_3035 |  | Hypothetical protein | | 7.2e^-41^ (88.6) |  | 1.9e^-22^ (54.6) | 1.0e^-23^ (56.7) | 4.0e^-22^ (64.6) | 4.0e^-26^ (63.3) | 1.0e^-24^ (55.6) |

| C4K02_3034 | *treY* | Maltooligosyl trehalose synthase (EC 5.4.99.15) | | 0.0 (89.2) | 0.0 (89.5) | 0.0 (72.5) | 0.0 (72.4) | 0.0 (72.4) | 0.0 (70.6) | | 0.0 (74.8) |
| --- | --- | --- | --- | --- | --- | --- | --- | --- | --- | --- | --- |
| C4K02_3033 | *malQ* | 4-α-glucanotransferase (EC 2.4.1.25) | | 0.0 (91.4) | 0.0 (89.8) | 0.0 (73.6) | 0.0 (75.1) | 0.0 (74.8) | 0.0 (73.6) | | 0.0 (74.5) |
| C4K02_3032 | *treZ* | Maltooligosyl trehalose hydrolase (EC 3.2.1.141) | | 0.0 (88.0) | 0.0 (86.2) | 0.0 (70.1) | 0.0 (71.6) | 0.0 (71.7) | 0.0 (69.0) | | 0.0 (69.6) |
| C4K02_3031 | *glgA* | Starch synthase (EC 2.4.1.21) | | 0.0 (96.3) | 0.0 (94.9) | 0.0 (86.1) | 0.0 (85.4) | 0.0 (85.1) | 0.0 (87.7) | | 0.0 (84.3) |
| Pathway: biosynthesis of N-acetylglutaminyleglutamine amide (NAGGN) | | | | | | | | | | | |
| C4K02_3962 | *ggnA* | | Asn synthase (Gln-hydrolyzing) (EC 6.3.5.4) | 0.0 (98.1) | 0.0 (98.1) | 0.0 (94.7) | 0.0 (93.6) | 0.0 (93.7) | 0.0 (94.2) | 0.0 (92.0) | |
| C4K02_3963 | *ggnB* | | GNAT-family acetyltransferase | 0.0 (95.2) | 0.0 (96.0) | 0.0 (89.3) | 0.0 (88.0) | 0.0 (87.6) | 0.0 (88.4) | 0.0 (87.4) | |
| C4K02_3964 |  | | Glutamyl aminopeptidase/ M42 family hydrolase | 0.0 (99.2) | 0.0 (98.0) | 0.0 (95.7) | 0.0 (92.6) | 0.0 (93.4) | 0.0 (94.4) | 0.0 (94.7) | |
| C4K02_3965 |  | | Hypothetical protein | 8.2e^-38^ (97.3) | 2.9e^-38^ (98.7) | 1.2e^-38^ (100) | 1.7e^-37^ (97.3) | 1.7e^-37^ (96.0) | 6.7e^-35^ (90.7) | 7.4e^-36^ (93.3) | |
| Pathway: biosynthesis of L-ectoine | | | | | | | | | | | |
| C4K02_0192 |  | | Diaminobutyrate-2-oxoglutarate transaminase (EC 2.6.1.76) |  | 0.0 (87.2) |  |  |  | 0.0 (49.0) | |  |
| C4K02_0193 |  | | Acetyltransferase (GNAT) family protein |  | 0.0 (96.1) |  |  |  | 0.0 (41.7) | |  |
| C4K02_0194 | *ectC* | | L-ornithine N5-oxygenase (EC 1.13.12.-) |  | 0.0 (92.4) |  |  |  | 0.0 (47.7) | |  |

| Pathway: uptake of quaternary amine osmoprotectants | | | | | | | | | |  |
| --- | --- | --- | --- | --- | --- | --- | --- | --- | --- | --- |
| C4K02_1863 | *betX* | Glycine betaine/proline transport system substrate-binding protein | 0.0 (97.9) | 0.0 (97.2) | 0.0 (87.2) | 0.0 (86.3) | 0.0 (87.0) | 0.0 (95.1) | 0.0 (92.6) | |
| C4K02_0262 | *betT2* | BCCT family transporter | 0.0 (98.0) | 0.0 (97.9) | 0.0 (93.5) | 0.0 (94.1) | 0.0 (93.7) | 0.0 (92.8) | 0.0 (92.8) | |
| C4K02_5542 | *betT3* | BCCT family transporter |  |  |  |  |  |  |  | |
| C4K02_5476 | *betA* | Choline dehydrogenase (EC 1.1.99.1) | 0.0 (99.3) | 0.0 (98.8) | 0.0 (95.2) | 0.0 (95.4) | 0.0 (94.7) | 0.0 (94.2) | 0.0 (95.4) | |
| C4K02_5475 | *betB* | Betaine aldehyde dehydrogenase (EC 1.2.1.8) | 0.0 (98.8) | 0.0 (98.6) | 0.0 (94.3) | 0.0 (93.3) | 0.0 (93.7) | 0.0 (94.9) | 0.0 (95.9) | |

| C4K02_5474 | *betI* | TetR family transcriptional regulator BetI | 0.0 (98.0) | 0.0 (93.9) | 0.0 (90.4) | 0.0 (88.7) | 0.0 (88.7) | 0.0 (87.7) | 0.0 (90.8) |
| --- | --- | --- | --- | --- | --- | --- | --- | --- | --- |
| C4K02_5472 | *betT1* | BCCT family transporter | 0.0 (98.2) | 0.0 (98.2) | 0.0 (92.0) |  |  | 0.0 (91.0) | 0.0 (92.5) |
| C4K02_5471 | *cbcV* | Glycine betaine/proline transport system ATP-binding protein | 0.0 (99.2) | 0.0 (97.2) | 0.0 (94.1) | 0.0 (94.4) | 0.0 (94.1) | 0.0 (94.4) | 0.0 (94.9) |
| C4K02_5470 | *cbcW* | Glycine betaine/proline transport system permease protein | 0.0 (99.6) | 0.0 (98.9) | 0.0 (95.4) | 0.0 (95.7) | 0.0 (95.7) | 0.0 (95.4) | 0.0 (96.1) |
| C4K02_5469 | *cbcX* | Glycine betaine/proline transport system substrate-binding protein | 0.0 (96.5) | 0.0 (95.9) | 0.0 (82.9) | 0.0 (83.2) | 0.0 (83.5) | 0.0 (86.7) | 0.0 (87.0) |
| C4K02_5467 | *gbdR* | AraC family transcriptional regulator GbdR | 0.0 (99.0) | 0.0 (99.5) | 0.0 (97.5) | 0.0 (97.5) | 0.0 (97.5) | 0.0 (97.0) | 0.0 (96.7) |
| C4K02_0892 | *opuA* | Osmoprotectant transport system permease protein | 0.0 (99.2) | 0.0 (99.6) | 0.0 (93.7) | 0.0 (92.9) | 0.0 (93.3) | 0.0 (92.9) | 0.0 (92.4) |
| C4K02_0891 | *opuB* | Osmoprotectant transport system substrate-binding protein | 0.0 (92.3) | 0.0 (94.6) | 0.0 (84.8) | 0.0 (84.1) | 0.0 (85.1) | 0.0 (91.2) | 0.0 (83.8) |

| C4K02_0890 | *opuC* | Osmoprotectant transport system permease protein | 0.0 (96.3) | 0.0 (99.1) | 0.0 (95.9) | 0.0 (95.9) | 0.0 (95.4) | 0.0 (98.2) | 0.0 (94.4) |
| --- | --- | --- | --- | --- | --- | --- | --- | --- | --- |
| C4K02_0889 | *opuD* | Osmoprotectant transport system ATP-binding protein | 0.0 (99.2) | 0.0 (97.9) | 0.0 (95.9) | 0.0 (94.5) | 0.0 (94.3) | 0.0 (94.3) | 0.0 (95.8) |
| Pathway: catabolism of quaternary amines – conversion of carnitine to glycine betaine | | | | | | | | | |
| C4K02_5465 | *cdhB* | Acyl-CoA thioester hydrolase (EC 3.1.2.-) | 0.0 (86.0) | 0.0 (89.7) | 0.0 (79.4) | 0.0 (75.6) | 0.0 (78.7) | 0.0 (77.4) | 0.0 (77.4) |
| C4K02_5464 | *cdhA* | L-carnitine dehydrogenase (EC 1.1.1.108) | 0.0 (95.6) | 0.0 (96.0) | 0.0 (94.4) | 0.0 (94.4) | 0.0 (94.1) | 0.0 (93.7) | 0.0 (95.3) |
| C4K02_5463 | *cdhC* | Hypothetical protein (DUF849 family) | 0.0 (96.9) | 0.0 (98.0) | 0.0 (96.6) | 0.0 (95.3) | 0.0 (94.2) | 0.0 (95.9) | 0.0 (95.3) |
| C4K02_5462 | *cdhX* | Glycine betaine/proline transport system substrate-binding protein | 0.0 (92.3) | 0.0 (93.9) | 0.0 (87.6) | 0.0 (85.3) | 0.0 (85.6) | 0.0 (86.0) | 0.0 (87.9) |

| C4K02_5461 | *cdhR* | AraC family transcriptional regulator CdhR | 0.0 (96.5) | 0.0 (95.9) | 0.0 (91.4) | 0.0 (88.5) | 0.0 (89.2) | 0.0 (89.7) | 0.0 (89.1) |
| --- | --- | --- | --- | --- | --- | --- | --- | --- | --- |
| Pathway: catabolism of quaternary amines – uptake and conversion of choline-*O*-sulfate to choline | | | | | | | | | |
| C4K02_0035 | *cosE* | ABC transporter permease, sulfate permease family | 0.0 (92.8) | 0.0 (94.6) | 0.0 (83.7) | 0.0 (83.0) | 0.0 (82.8) | 0.0 (82.2) | 0.0 (83.7) |
| C4K02_0036 | *cosX* | Glycine betaine/proline transport system substrate-binding protein | 0.0 (90.8) | 0.0 (91.8) | 0.0 (87.6) | 0.0 (87.6) | 0.0 (87.6) | 0.0 (81.7) | 0.0 (85.5) |
| C4K02_0037 | *cosC* | Choline-sulfatase (EC 3.1.6.6) | 0.0 (97.0) | 0.0 (96.4) | 0.0 (91.9) | 0.0 (92.7) | 0.0 (91.9) | 0.0 (91.7) | 0.0 (92.7) |
| C4K02_0038 | *cosR* | LysR family transcriptional regulator CosR | 0.0 (90.2) | 0.0 (93.4) | 0.0 (79.1) | 0.0 (78.8) | 0.0 (79.4) | 0.0 (78.1) | 0.0 (78.8) |

| Pathway: catabolism of quaternary amines – conversion of dimethyl glycine to sarcosine | | | | | | | | | |
| --- | --- | --- | --- | --- | --- | --- | --- | --- | --- |
| C4K02_5456 |  | Membrane dipeptidase (EC 3.4.13.19) | 0.0 (99.1) | 0.0 (98.8) | 0.0 (95.4) | 0.0 (98.2) | 0.0 (97.8) | 0.0 (93.5) | 0.0 (94.2) |
| C4K02_5455 |  | Hypothetical protein | 0.0 (98.9) | 0.0 (98.3) | 0.0 (98.9) | 0.0 (98.3) | 0.0 (98.3) | 0.0 (98.3) | 0.0 (98.9) |
| C4K02_5454 | *dgcA* | 2,4-dienoyl-CoA reductase-like NADH-dependent reductase (dimethylglycine demethylase) | 0.0 (99.0) | 0.0 (98.1) | 0.0 (96.1) | 0.0 (97.7) | 0.0 (97.4) | 0.0 (95.9) | 0.0 (97.1) |
| C4K02_5453 | *dgcB* | Fe-S oxidoreductase | 0.0 (97.4) | 0.0 (96.6) | 0.0 (91.1) | 0.0 (92.1) | 0.0 (92.0) | 0.0 (91.8) | 0.0 (92.0) |
| C4K02_5452 |  | Electron transfer flavoprotein, α subunit | 0.0 (95.8) | 0.0 (96.1) | 0.0 (91.4) | 0.0 (91.4) | 0.0 (91.9) | 0.0 (88.8) | 0.0 (90.4) |
| C4K02_5451 |  | Electron transfer flavoprotein, β subunit | 0.0 (92.7) | 0.0 (91.8) | 0.0 (83.6) | 0.0 (82.8) | 0.0 (82.4) | 0.0 (83.6) | 0.0 (85.5) |
| Pathway: catabolism of quaternary amines – conversion of glycine betaine to dimethyl glycine | | | | | | | | | |
| C4K02_5449 | *gbcA* | Rieske 2Fe-2S family protein | 0.0 (98.8) | 0.0 (98.8) | 0.0 (96.5) | 0.0 (97.2) | 0.0 (97.7) | 0.0 (95.6) | 0.0 (95.3) |

| C4K02_5448 | *gbcB* | Ferredoxin-NADP reductase | 0.0 (97.8) | 0.0 (98.6) | 0.0 (94.0) | 0.0 (94.8) | 0.0 (96.7) | 0.0 (95.4) | 0.0 (95.1) |
| --- | --- | --- | --- | --- | --- | --- | --- | --- | --- |
| Pathway: catabolism of quaternary amines – conversion of sarcosine to glycine | | | | | | | | | |

| C4K02_2979 | *souR* | AraC-type sarcosine catabolism regulator SouR | 0.0 (93.5) | 0.0 (91.1) | 0.0 (86.6) |  |  |  | 0.0 (80.1) |
| --- | --- | --- | --- | --- | --- | --- | --- | --- | --- |
| C4K02_5443 | *glyA2* | Gly/Ser hydroxymethyl-transferase (EC 2.1.2.1) | 0.0 (98.8) | 0.0 (97.8) | 0.0 (96.6) | 0.0 (96.6) | 0.0 (96.4) | 0.0 (95.7) | 0.0 (96.6) |

| C4K02_5442 | *soxB* | Sarcosine oxidase, β subunit | 0.0 (100) | 0.0 (100) | 0.0 (99.3) | 0.0 (99.3) | 0.0 (99.0) | 0.0 (97.8) | 0.0 (98.8) |  |
| --- | --- | --- | --- | --- | --- | --- | --- | --- | --- | --- |
| C4K02_5441 | *soxD* | Sarcosine oxidase, δ subunit | 0.0 (98.1) | 0.0 (97.0) | 0.0 (90.9) | 0.0 (90.9) | 0.0 (91.9) | 0.0 (97.0) | 0.0 (91.9) |  |
| C4K02_5440 | *soxA* | Sarcosine oxidase, α subunit | 0.0 (97.9) | 0.0 (97.8) | 0.0 (94.1) | 0.0 (94.5) | 0.0 (94.8) | 0.0 (94.9) | 0.0 (94.7) |  |
| C4K02_5439 | *soxG* | Sarcosine oxidase, γ subunit | 0.0 (94.3) | 0.0 (93.8) | 0.0 (92.8) | 0.0 (90.4) | 0.0 (90.9) | 0.0 (90.9) | 0.0 (87.6) |  |
| C4K02_5438 | *purU* | Formyltetrahydrofolate deformylase (EC 3.5.1.10) | 0.0 (99.6) | 0.0 (99.3) | 0.0 (96.1) | 0.0 (96.8) | 0.0 (96.5) | 0.0 (94.0) | 0.0 (93.3) |  |
| C4K02_5437 | *fdhA1* | Glutathione-independent formaldehyde dehydrogenase (EC 1.2.1.46) | 0.0 (98.2) | 0.0 (99.2) | 0.0 (97.5) | 0.0 (98.2) | 0.0 (99.5) | 0.0 (95.5) | 0.0 (96.5) |  |
| Pathway: synthesis of alginate | | | | | | | | | | |
| C4K02_1064 | *algD* | GDP-mannose 6-dehydrogenase (EC 1.1.1.132) | 0.0 (97.9) | 0.0 (97.5) | 0.0 (94.5) | 0.0 (94.1) | 0.0 (93.8) | 0.0 (93.8) | 0.0 (94.7) |  |
| C4K02_1063 | *algB* | Alginate biosynthesis protein AlgB | 0.0 (98.4) | 0.0 (98.0) | 0.0 (92.7) | 0.0 (92.5) | 0.0 (92.7) | 0.0 (92.9) | 0.0 (93.9) |  |
| C4K02_1062 | *alg44* | Alginate biosynthesis protein Alg44 | 0.0 (99.2) | 0.0 (95.9) | 0.0 (85.1) | 0.0 (83.0) | 0.0 (82.0) | 0.0 (83.8) | 0.0 (85.3) |  |
| C4K02_1061 | *algK* | Alginate biosynthesis protein AlgK | 0.0 (98.3) | 0.0 (96.5) | 0.0 (85.0) | 0.0 (82.8) | 0.0 (81.8) | 0.0 (87.1) | 0.0 (86.1) |  |
| C4K02_1060 | *algE* | Alginate export protein AlgE | 0.0 (98.0) | 0.0 (94.5) | 0.0 (85.1) | 0.0 (82.3) | 0.0 (82.4) | 0.0 (86.1) | 0.0 (81.0) |  |

| C4K02_1059 | *algG* | Poly(beta-D-mannuronate) C5 epimerase (EC 5.1.3.-) | 0.0 (97.9) | 0.0 (97.5) | 0.0 (94.5) | 0.0 (94.1) | 0.0 (93.8) | 0.0 (93.8) | 0.0 (94.7) |
| --- | --- | --- | --- | --- | --- | --- | --- | --- | --- |
| C4K02_1058 | *algX* | Alginate export protein AlgX | 0.0 (98.9) | 0.0 (97.5) | 0.0 (90.1) | 0.0 (86.3) | 0.0 (85.9) | 0.0 (87.0) | 0.0 (88.4) |

| C4K02_1057 | *algL* | Poly(beta-D-mannuronate) lyase precursor (EC 4.2.2.3) | 0.0 (96.3) | 0.0 (93.2) | 0.0 (86.7) | 0.0 (83.5) | 0.0 (82.9) | 0.0 (82.0) | 0.0 (83.7) |  |
| --- | --- | --- | --- | --- | --- | --- | --- | --- | --- | --- |
| C4K02_1056 | *algI* | Alginate O-acetyltransferase complex protein AlgI (EC 2.3.1.-) | 0.0 (98.8) | 0.0 (98.6) | 0.0 (90.4) | 0.0 (90.4) | 0.0 (90.4) | 0.0 (91.0) | 0.0 (90.8) |  |
| C4K02_1055 | *algJ* | Alginate O-acetyltransferase complex protein AlgJ | 0.0 (95.2) | 0.0 (95.4) | 0.0 (82.9) | 0.0 (81.2) | 0.0 (80.9) | 0.0 (80.9) | 0.0 (81.6) |  |
| C4K02_1054 | *algF* | Alginate O-acetyl transferase complex protein AlgF | 0.0 (100.0) | 0.0 (95.4) | 0.0 (89.9) | 0.0 (87.8) | 0.0 (87.4) | 0.0 (86.5) | 0.0 (90.8) |  |
| C4K02_1053 | *algA* | mannose-1-phosphate guanylyltransferase (GDP) /mannose-6-phosphate isomerase, type 2 | 0.0 (98.1) | 0.0 (97.3) | 0.0 (93.0) | 0.0 (94.4) | 0.0 (94.6) | 0.0 (94.4) | 0.0 (94.0) |  |
| Pathway: synthesis of the Psl exopolysaccharide | | | | | | | | | | |
| C4K02_2009 | *pslA* | Undecaprenyl-phosphate glucose phosphotransferase | 0.0 (87.4) | 0.0 (83.1) | 0.0 (36.6) | 3.8e^-29^ (38.8) | 5.4e^-28^ (38.0) | 0.0 (61.3) | 0.0 (74.9) |  |
| C4K02_2008 | *pslB* | mannose-1-phosphate guanylyltransferase /mannose-6-phosphate isomerase | 0.0 (92.7) | 0.0 (87.4) | 0.0 (58.3) | 0.0 (57.5) | 0.0 (57.7) | 0.0 (67.4) | 0.0 (77.2) |  |
| C4K02_2007 | *pslC* | rhamnosyltransferase | 0.0 (77.4) | 0.0 (83.8) |  |  |  | 0.0 (52.1) | 0.0 (76.9) |  |
| C4K02_2006 | *pslD* | polysaccharide export protein |  | 0.0 (42.2) |  |  |  |  | 0.0 (78.5) |  |
| C4K02_2005 | *pslE* | uncharacterized exopolysaccharide biosynthesis protein |  | 0.0 (54.1) |  |  |  | 0.0 (48.6) | 0.0 (78.3) |  |

| C4K02_2004 | *pslF* | glycosyltransferase cell wall biosynthesis | 0.0 (64.2) | 0.0 (66.2) |  |  |  | 0.0 (63.3) | 0.0 (82.8) |
| --- | --- | --- | --- | --- | --- | --- | --- | --- | --- |
| C4K02_2003 | *pslG* | beta-xylosidase | 0.0 (53.5) | 0.0 (51.7) |  |  |  | 0.0 (51.3) | 0.0 (76.4) |
| C4K02_2002 | *pslH* | glycosyltransferase cell wall biosynthesis |  | 0.0 (57.0) |  |  |  | 0.0 (61.2) | 0.0 (75.7) |
| C4K02_2001 | *pslI* | glycosyltransferase cell wall biosynthesis |  | 0.0 (53.3) |  | 4.1e^-23^ (31.6) | 4e^-23^ (31.6) | 0.0 (55.2) | 0.0 (72.9) |
| C4K02_2000 | *pslJ* | O-antigen ligase-like membrane protein |  | 0.0 (53.9) |  |  |  | 0.0 (54.6) | 0.0 (83.5) |
| C4K02_1999 |  | acetyltransferase-like isoleucine enzyme |  |  |  |  |  | 0.0 (59.9) | 0.0 (72.9) |
| C4K02_1998 | *pslK* | peptidoglycan biosynthesis protein MviN/MurJ (putative lipid II flippase) |  | 0.0 (64.7) |  |  |  | 0.0 (66.4) | 0.0 (76.9) |
